# A bibliometric and visualized analysis of extracellular vesicles in degenerative musculoskeletal diseases (from 2006 to 2024)

**DOI:** 10.3389/fphar.2025.1550208

**Published:** 2025-03-13

**Authors:** Jun-Jie Yang, Sha-Qi He, Bei Huang, Peng-Xin Wang, Feng Xu, Xiao Lin, Jun Liu

**Affiliations:** ^1^ Department of Radiology, The Second Xiangya Hospital, Central South University, Changsha, China; ^2^ Department of Radiology, The Second Affiliated Hospital of Xinjiang Medical University, Ürümqi, China; ^3^ National Clinical Research Center for Metabolic Diseases, Department of Metabolism and Endocrinology, The Second Xiangya Hospital, Central South University, Changsha, China; ^4^ Clinical Research Center for Medical Imaging in Hunan Province, Department of Radiology Quality Control Center in Hunan Province, Changsha, China

**Keywords:** degenerative musculoskeletal diseases, bibliometric analysis, extracellular vesicles, senescence, cell-free therapy

## Abstract

**Background:**

With the rapid development of extracellular vesicles (EVs) in regenerative medicine research, they have become a promising new direction in the mechanistic, diagnosis and treatment studies of degenerative musculoskeletal diseases (DMDs), and has attracted increasing attention. However, there is currently a lack of comprehensive and objective summary analysis to help researchers quickly and conveniently understand the development trajectory and future trends of this field.

**Method:**

This study collected articles and reviews published from 2006 to 2024 on EVs in DMDs from the Web of Science database. Bibliometric and visual analysis was conducted using several tools, including Microsoft Excel Office, VOSviewer, CiteSpace, Pajek, and R packages.

**Results:**

1,182 publications were included in the analysis from 2006 to 2024. Notably, there was a rapid increase in the number of publications starting in 2016, suggesting that this field remains in a developmental stage. Co-authorship analysis revealed that China ranked first in terms of publications, whereas the United States led in citations. The journal with the highest number of publications was International Journal of Molecular Sciences (INT J MOL SCI). The most prolific authors were Ragni, E with 23 publications, while the most cited author was Toh, WS. Additionally, nine of the top 10 institutions were from China, with Shanghai Jiao Tong University leading in the number of publications. The most cited article was “MSC exosomes mediate cartilage repair by enhancing proliferation, attenuating apoptosis and modulating immune reactivity”, authored by Zhang, S, and published in BIOMATERIALS in 2018.

**Conclusion:**

This study, through bibliometric and visual analysis, clearly illustrates the collaborative relationships among countries, authors, institutions, and journals, providing valuable insights for researchers seeking academic collaboration opportunities. Moreover, the analysis of keywords and citations allows researchers to better understand key research hotspots and frontiers in this field, and points toward promising directions for future research. The growing interest in EV research in DMDs over recent years indicates increasing attention and a dynamic progression in this field.

## 1 Introduction

Degenerative musculoskeletal diseases (DMDs) involve structural and functional impairments of the musculoskeletal system, leading to symptoms such as pain and restricted movement, which significantly impact patients’ quality of life (Wen et al., 2023; Yin et al., 2023). Common types of DMDs include osteoarthritis (OA) (Hunter and Bierma-Zeinstra, 2019), osteoporosis (OP) (Compston et al., 2019), degenerative disc disease (DDD) (Xiang et al., 2024), and sarcopenia (Cruz-Jentoft and Sayer, 2019). As life expectancy rises and the global population ages, these DMDs have led to substantial economic and societal burdens (Scheuing et al., 2023; Clynes et al., 2020; Kirk et al., 2020). The primary treatment for OP focuses on pharmacological interventions, including calcium, vitamin D, estrogen receptor modulator, RANKL receptor agonists, and parathyroid hormone analogs (LeBoff et al., 2022; Reid and Billington, 2022). Nevertheless, several drugs cause serious adverse reactions or are not suitable for prolonged use (Song et al., 2022). For OA, there is currently no cure. Treatments mainly aim to relieve pain and maintain joint function. Nonsteroidal anti-inflammatory drugs (NSAIDs) and acetaminophen are typically first-line therapies, while total joint replacement surgery is considered the gold standard for patients with severe OA (not respond to conservative treatments or the quality of life severely affected by pain) (Abramoff and Caldera, 2020). Treatment for sarcopenia focuses on avoiding mobility impairment and significant health events like fractures, primarily through strength exercise and enhanced dietary energy intake (Chen et al., 2022; Coletta and Phillips, 2023; Dent et al., 2018). DDD can only alleviate pain through physical therapy, medications, and surgical intervention (Zhu et al., 2023). However, there is a high risk of recurrence and surgical complications. However, they do not address the underlying causes. This highlights the need for more direct and effective treatment strategies that can reverse the disease by eliminating pathogenic factors and promoting tissue repair.

Extracellular vesicles (EVs) are nanoscale lipid bilayer structures derived from cells that encapsulate proteins, lipids, RNA, metabolites, growth factors, and cytokines (Weng et al., 2021). EVs play multiple roles in intercellular communication by transporting their cargo to recipient cells, where they can modulate cellular functions or physiological activities, and even affect organs (Jeppesen et al., 2023). Additionally, EVs may contribute to the emergence of several diseases, such as cancer (Ciardiello et al., 2016) and neurological disorders (Izquierdo-Altarejos et al., 2024), by transporting abnormal cargo. The pivotal role of EVs in intercellular communication positions them as promising candidates for therapeutic applications, drug delivery systems, and disease biomarkers (Kumar M. A. et al., 2024). As a drug delivery system, EVs offer advantages over cell therapies, including low immunogenicity, high biocompatibility, low oncogenicity, the capacity to traverse biological barriers such as the blood-brain barrier, and a lipid membrane structure that protects enclosed bioactive molecules (Kou et al., 2022; Elsharkasy et al., 2020; Vader et al., 2016). Moreover, EVs can be engineered to modify their surfaces (Chen Z. et al., 2024; Zhang et al., 2023) and combined with biomaterials (Yang et al., 2024; Yin et al., 2022; Liao et al., 2022; Fang et al., 2023) to enhance their targeting and retention time at the target. Interestingly, the use of EVs as drug delivery vehicles for treating DMDs is gaining increasing research attention.

In addition, EVs are increasingly recognized for their potential as biomarkers in disease diagnosis, particularly for early cancer detection (Kumar M. A. et al., 2024; Kalluri, 2016; Wang et al., 2022). Although EVs as a diagnostic tool for DMDs are still in the preclinical stage, it is potential to replace conventional clinical diagnostics in the future, truly enabling early diagnosis and treatment.

Bibliometrics involves analyzing abstracts, keywords, and citations of published papers, using statistical data to describe or display relationships among published papers (Ninkov et al., 2022). While numerous high-quality reviews on EVs in the field of DMDs have been published, traditional literature review methods have become inadequate for researchers to track the state, development, and evolution, to identify gaps, and to categorize the knowledge system (Öztürk et al., 2024). Bibliometric analysis addresses this limitation by considering all literatures related to the research, revealing the scientific trends in different fields and providing insights into ongoing discussions about evaluating science and its researchers (Ellegaard, 2018). Therefore, this study primarily utilizes data visualization tools, including Citespace and VOSviewer, to perform bibliometric and visual analyses of EVs research in DMDs over the last 18 years. This study aims to provide new perspectives for future research, allowing researchers to easily access the latest trends and developments in the field, and offering useful references for possible collaborations.

## 2 Materials and methods

### 2.1 Data sources and search strategies

**FIGURE 1:**
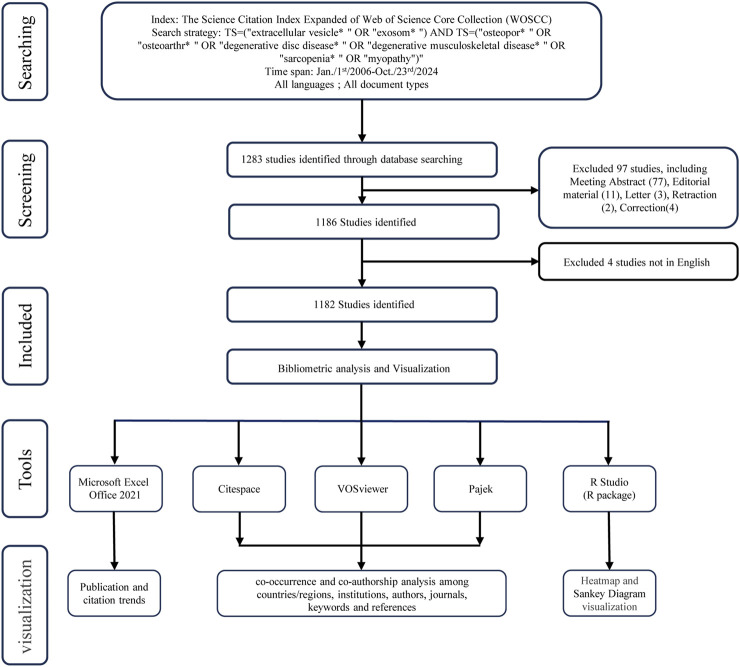
Flowchart of literature search and selection.

The bibliometric data for this study were sourced from the Web of Science Core Collection (WoSCC), a widely recognized and standardized academic database. We retrieved all online articles and reviews on EVs in DMDs published between 2006 and 2024 from the WoSCC SCI-expanded database. The search strategy employed was: “TS=(“extracellular vesicle* “OR” exosom*”) AND TS=(“osteopor* “OR” osteoarthr*” OR “degenerative disc disease* “OR” degenerative musculoskeletal disease* “OR” sarcopenia* “OR” myopathy”)”. The search interval was from 1 January 2006 to 23 October 2024. The document types chosen were limited to “Article” and “Review,” with English as the language. In total, 1,182 original articles and reviews met the criteria for inclusion in the analyses, and were further analyzed visually (Figure 1).

### 2.2 Data analysis and visualization

The comprehensive data was collected from WoSCC and loaded into Microsoft Excel Office 2021 (Microsoft Corporation, Redmond, United States), VOSviewer 1.6.20 (Leiden University, Netherlands), Citespace version 6.3. R1/6.2. R7 (Chaomei Chen, China), Pajek version 5.19 (University of Ljubljana, Slovenia), GraphPad Prism version 10.1.2 (GraphPad Software, San Diego, California United States, www.graphpad.com), and the chorddiag R package (R Studio, version 4.3.2). VOSviewer and Pajek were utilized to conduct co-occurrence and co-authorship analysis to identify patterns of co-occurrence and co-authorship among countries/regions, institutions, authors, journals, keywords. Citespace was utilized for visualization, allowing the creation of visual maps related to keywords, co-cited references, journals, and citation bursts (Chen, 2004; Chen et al., 2010; Chen, 2006). Bubble chart was plotted by https://www.bioinformatics.com.cn, an online platform for data analysis and visualization (Tang et al., 2023). Additionally, Bibliometrix package in R software (Aria and Cuccurullo, 2017) was used for visual analysis of counties/regions, authors and keywords.

## 3 Results

### 3.1 Analysis of annual publications

**FIGURE 2:**
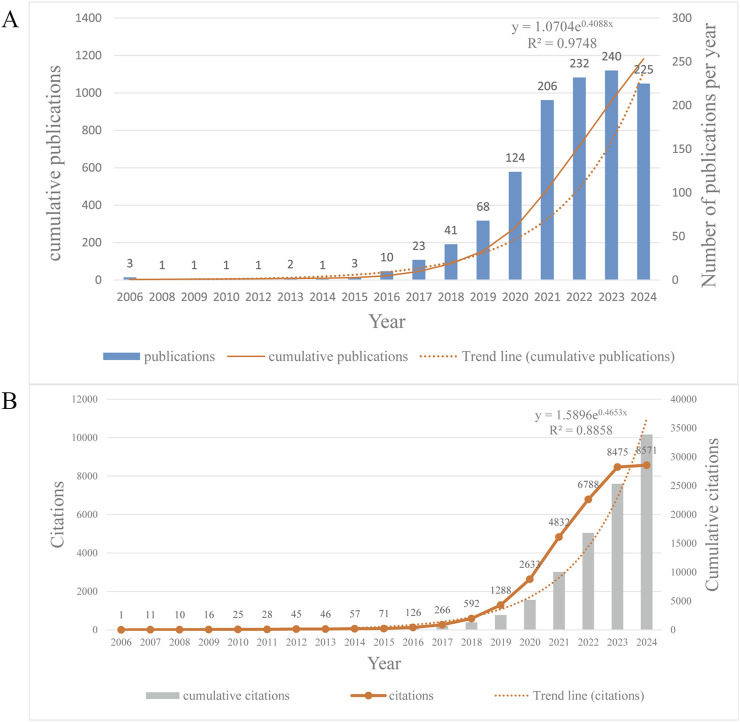
**(A)** Annual and cumulative publications from 2006 to 2024, and the trend line of annual publications. It should be noted that the articles we analyzed were published up to 23 October 2024, so the publications of this year are incomplete and lower compared to the previous year, but the prediction still shows an upward trend. **(B)** Annual and cumulative citations from 2006 to 2024, and the trend line of annual citations.

We collected 1,182 relevant documents, including reviews and articles, from 1 January 2006 to 23 October 2024 for the study of EVs in DMDs (Figure 2). The number of scholarly publications in this field has shown a steady increase over the past decade (Figure 2A). Prior to 2016, the volume of publications in this field was modest, suggesting that Scholarly exploration in this area had only just begun. However, the number of publications began to rise significantly after 2016, reaching a peak in 2023 with 240 annual publications, marking a 24-fold increase compared to 2016. However, the total number of publications on this topic remains relatively modest, with only 1,182 cumulative publications as of November 2024. Nevertheless, the high growth rate suggests promising prospects for the development of this field. To project future trends, we apply the exponential equation y = 1.0704^0.4088x^ to model the anticipated trajectory of publication growth. The *R* ^2^ value of 0.9748 reflects the model’s excellent predictive ability, allowing for accurate forecasting of future publication trends. The prediction curve indicates a consistent rise in the annual number of articles, along with an increase in research activity in this field. Between 2006 and 2024, the total number of citations for all publications has reached 33,896, with the highest annual citation count occurring in 2024. On average, each publication has received 28.68 citations. The annual citation count exhibits an exponential growth trend, with particularly rapid increases observed since 2018, becoming even more pronounced after 2020. By 2023, the annual citations had reached 8,475. Based on the prediction model, the annual citations are expected to surpass 10,000 by the end of 2024. The exponential growth model (y = 1.5896e ^0.4653×^, *R* ^2^ = 0.8858) demonstrates a strong fit to the data, indicating that the upward trajectory of annual citations is likely to continue. This trend reflects the growing influence and increasing recognition of research in this field over time (Figure 2B).

### 3.2 Countries/regions analysis

**FIGURE 3:**
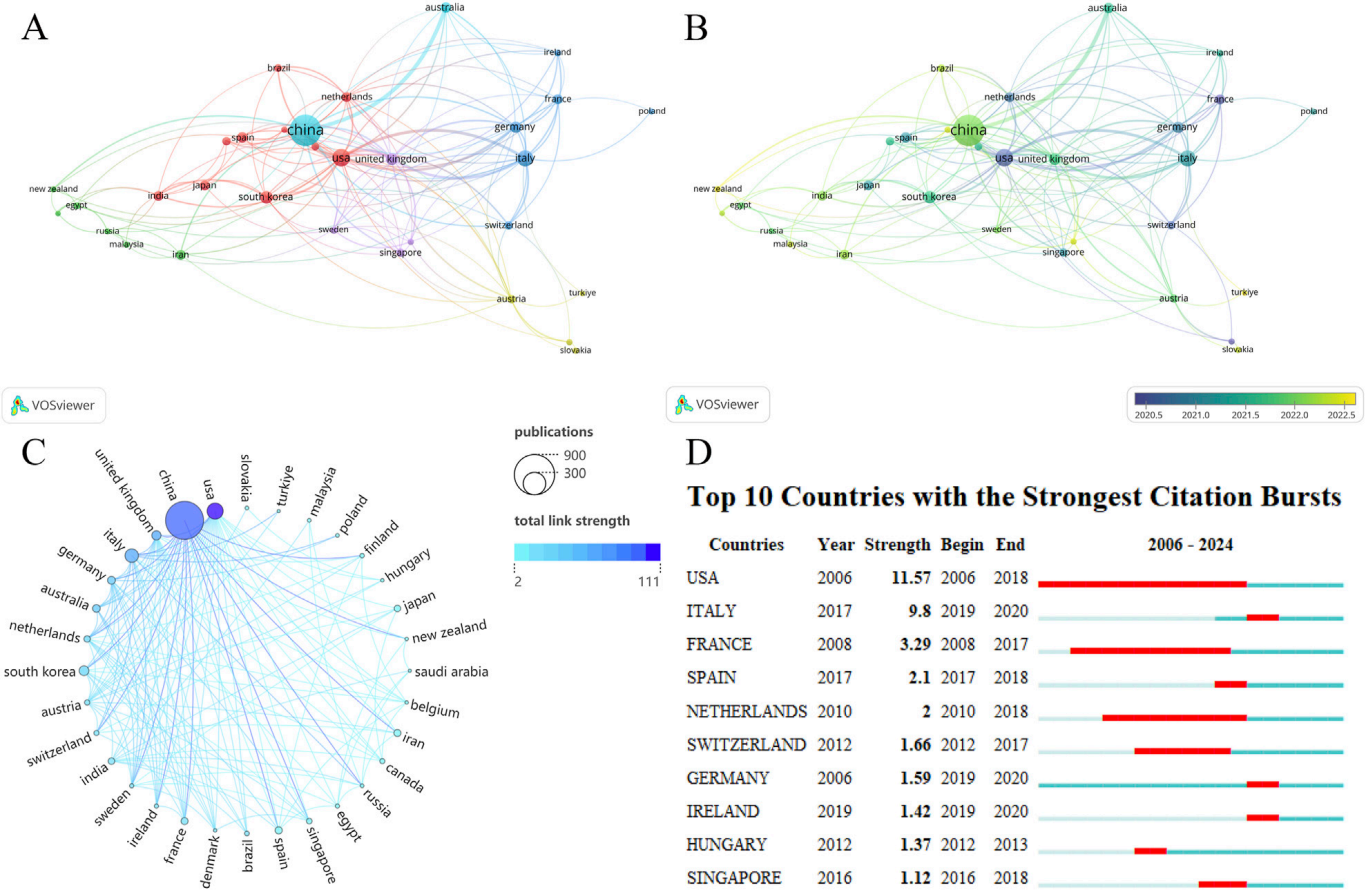
**(A)** Countries/regions network visualization. **(B)** Countries/regions overlay visualization. The size of the nodes also represents the publications of each country, and the color of the nodes reflects the average time of publication. **(C)** The chord diagram for countries/regions partnership. The United States has significantly fewer publications than China but has stronger cooperation with other countries and a higher Total link strength. **(D)** Top 10 countries with the strongest citation bursts.

EVs research in the field of DMDs involves 61 countries/regions. We screened 32 countries based on the requirement of at least five publications per country or region in DMDs, visualizing the number of publications in each country/region and the collaborations between countries/regions (Figure 3). As seen in Table 1, the top 10 countries/regions ranked by publications are China (n = 716), the United States (n = 132), and Italy (n = 92) respectively. Notably, although China significantly surpasses the United States in publications, its total link strength is lower than that of the United States, reflecting a lack of collaborative communication with other countries compared to the United States. This phenomenon can also be visually observed in Figure 3C. Statistical analysis revealed a significant correlation between the number of publications (NP) and total link strength, which represents the level of international collaboration among countries (P < 0.0001). Furthermore, countries were divided into high, medium, and low total link strength groups using tertile classification. A comparison of NP across these groups revealed that countries with high link strength published significantly more papers than the other two groups. Despite China achieving the highest publication output globally with relatively low total link strength, these findings underscore the critical role of international collaboration in accelerating research growth, as countries with stronger global partnerships tend to experience faster development in this domain (Supplementary Figures 1A, C). Most countries began research in this field after 2016, while a few, such as the United States, Germany, and France, started earlier and have established solid research foundations. However, the majority of publications have been concentrated in the past 3 years. Although China currently has a large number of publications, it is still in its early stages in this field, indicating a very rapid pace of development (Figures 3A, B; Supplementary Figure 2A). The national cooperation map offers a more intuitive visualization of collaborative relationships between countries (Supplementary Figure 2B). Figure 3D depicts the top 10 countries with the strongest citation bursts, with the United States showing both the highest intensity and the longest duration. This indicates that the U.S. has long been a leader in research hotspots and trends in this field. Overall, research in this field is still in its early stages globally, but growing interest suggests significant potential and promising prospects for future development.

**TABLE 1:** Top 10 Countries/regions ranked by publications with NC and total link strength.

Rank	Country/region	NP	NC	Total link strength
1	China	716	19739	94
2	United States	132	5211	111
3	Italy	92	2220	57
4	South Korea	48	1181	24
5	United Kingdom	37	573	61
6	Germany	31	935	42
7	Australia	30	846	36
8	Spain	26	721	11
9	France	25	1179	15
10	Iran	25	524	9

### 3.3 Keywords analysis

**FIGURE 4:**
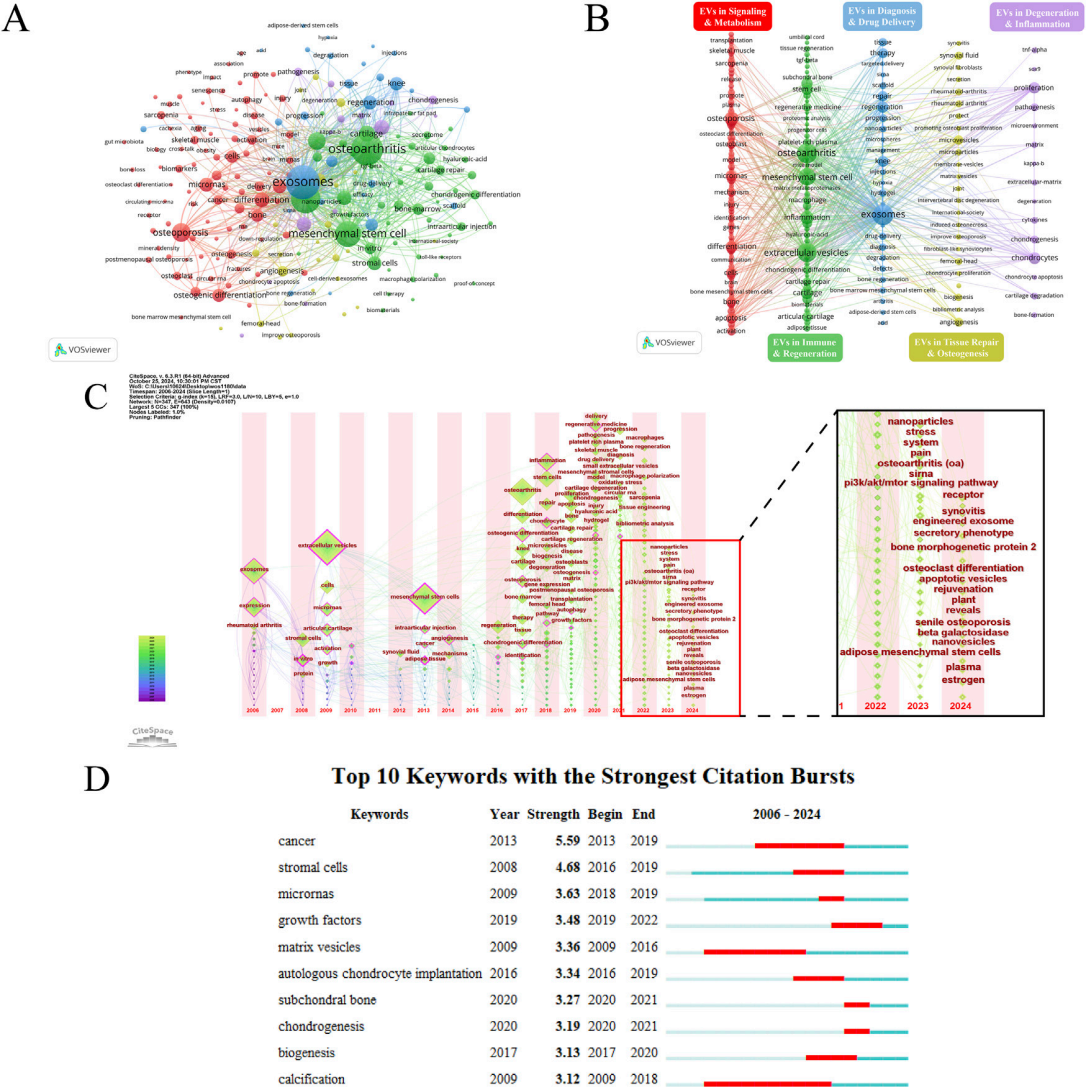
**(A)** Keyword network visualization. Circles and labels form the nodes, and the size of the node represents the frequency of the keyword, the higher the frequency, the larger the node. **(B)** Analysis of Keyword Clustering. The lines between the nodes represent the association strength between keywords. The color of the nodes represents their clusters, with different colors representing different clusters, divided into five clusters based on different research directions. **(C)** Temporal overlay visualization of the keyword co-occurrence network. The position of the nodes reflects the first appearance year of the corresponding keywords, the size of the nodes reflects the frequency of keyword occurrences, and the annual ring color reflects all years of keyword occurrence. The zoomed-in image shows the keywords that have emerged in the past 2 years. **(D)** Top 10 keywords with the strongest citation bursts.

Keywords often reflect the core content of an article. Through keyword co-occurrence analysis, research hotspots in a specific field can be identified. Using VOSviewer, a keyword co-occurrence network view was drawn for 1,182 publications, visualizing keywords with a frequency of eight or more (Figure 4). Table 2 shows the top 30 keywords by frequency. Figures 4A, B reveal the frequency of keywords and the results of cluster analysis, the top three keywords are exosomes, osteoarthritis and extracellular vesicles. Five clusters are generated which can represent the five main research directions in this field. The average year of keyword occurrence can be used to understand the frontiers and hotspots in this field (e.g., drug delivery, hydrogel, oxidative stress, nanoparticles, bone regeneration, macrophage polarization, sarcopenia, intervertebral disc degeneration, etc.) (Supplementary Figure 3A). The current high research interest in exosomes combined with new biomaterials makes exosomes and hydrogels appear close to each other in the density visualization (Supplementary Figure 3B). Many new keywords began to appear after 2016, and most of them were heavily cited during this period (Supplementary Figures 3C, D). This indicates that the field is an emerging research area, with continuous advancements and numerous new research directions, aligning with the surge in publications. Figure 4C illustrates the evolving research themes over time by tracking changes in keywords from 2006 to 2024. Figure 4D is the top 10 keywords with strongest citation burst, which reveals the changing trends of research hotspots in this academic field over time. It also highlights which keywords gained more citations during specific periods, reflecting the academic community’s focus on these topics.

**TABLE 2:** Top 30 keywords ranked by the frequency of occurrences, with total link strength.

Rank	Keyword	Occurrences	Total link strength
1	Exosomes	682	4,843
2	Osteoarthritis	607	4,428
3	Extracellular vesicles	522	3877
4	Mesenchymal stem cell	392	3110
5	Osteoporosis	211	1,309
6	Inflammation	182	1,465
7	Cartilage	151	1,205
8	Chondrocytes	135	1,030
9	Differentiation	134	962
10	Stem cell	129	1,033
11	Proliferation	128	989
12	Regeneration	118	933
13	Micrornas	118	866
14	Stromal cells	117	9,997
15	Apoptosis	109	798
16	Knee	106	809
17	Osteogenic differentiation	104	744
18	Cells	97	609
19	Bone	95	642
20	Repair	92	789
21	Articular-cartilage	85	695
22	*In-vitro*	81	667
23	Therapy	79	593
24	Bone-marrow	78	666
25	Microvesicles	67	583
26	Angiogenesis	63	459
27	Microrna	62	508
28	Osteoblast	62	495
29	Mechanism	59	454
30	Activation	58	392

### 3.4 Co-cited reference analysis

**FIGURE 5:**
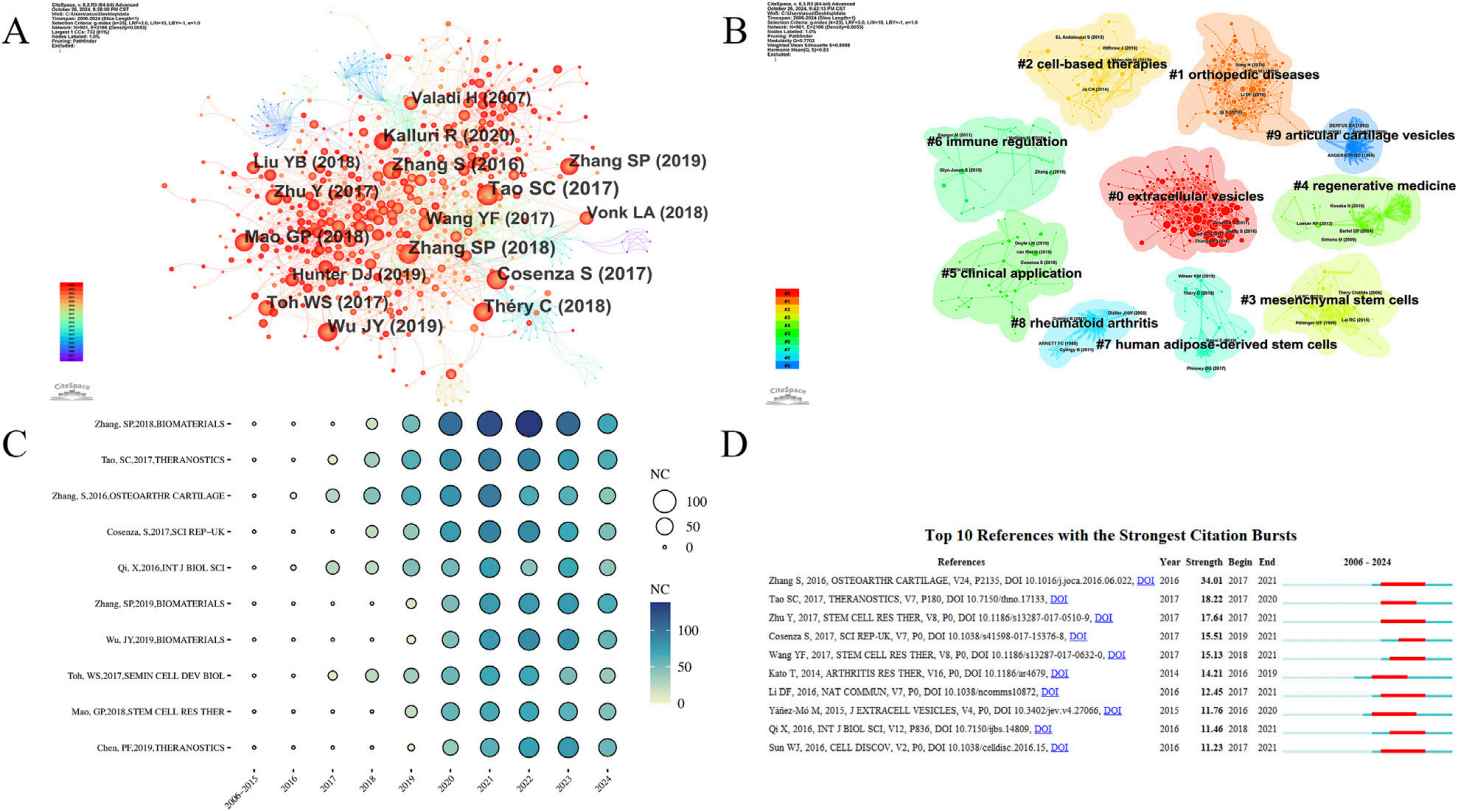
**(A)** Analysis of the network of references by citespace. Each node corresponds to each cited reference. The lines between nodes represent co-citation relationships. Various colors from purple to red represent different years, with the time range from 2006 to 2024. **(B)** The clustered network map of co-cited references. Different colors represent different clusters, which reflect the strength of association between articles. **(C)** Bubble chart of the top 10 cited references. The size and color of the bubbles intuitively show the citations of the articles and their trends over the years. **(D)** Top 10 references with the strongest citation bursts.

This section analyzes the field’s development process and the evolution of research hotspots through co-cited references and citation bursts. The co-cited reference analysis was conducted using CiteSpace (Figure 5). Table 3 lists the top 10 most-cited articles. These highly cited articles often reflect major discoveries and research achievements in the field at the time. Analyzing them provides valuable insights into the field’s developmental process. Figure 5A shows the cited articles and the co-citation relationships among them. In Figure 5B, each cluster represents a research topic or field, and the title of each cluster summarizes its main research topic. There are a total of 10 clusters: 0# (extracellular vesicles), 1# (orthopedic diseases), 2# (cell-based therapy), 3# (Mesenchymal stem Cells), 4# (regenerative medicine), 5# (clinical application), 6# (immune regulation), 7# (human adipose-derived stem cells), 8# (rheumatoid arthritis), 9# (articular cartilage vesicles). The two most active clusters are 0# (extracellular vesicles) and 1# (orthopedic disease), each containing over 100 keywords. As shown in Figure 5C, a sudden sharp increase in citations often indicates that the article has likely addressed a significant issue in the research field. Figure 5D shows the top 10 references in citation burst analysis. Most articles are heavily cited in 2016 ∼ 2018 when this research field was rapidly growing.

**TABLE 3:** Top 10 most-cited articles with journal, IF and total citation.

Rank	Year	Article	Journal	If (2023)	Total citation
1	2018	MSC exosomes mediate cartilage repair by enhancing proliferation, attenuating apoptosis and modulating immune reactivity	BIOMATERIALS	12.8	607
2	2017	Exosomes derived from miR-140-5p-overexpressing human synovial mesenchymal stem cells enhance cartilage tissue regeneration and prevent osteoarthritis of the knee in a rat model	THERANOSTICS	12.4	514
3	2016	Exosomes derived from human embryonic mesenchymal stem cells promote osteochondral regeneration	OSTEOARTHR CARTILAGE	7.2	482
4	2017	Mesenchymal stem cells derived exosomes and microparticles protect cartilage and bone from degradation in osteoarthritis	SCI REP-UK	3.8	429
5	2016	Exosomes Secreted by Human-Induced Pluripotent Stem Cell-Derived Mesenchymal Stem Cells Repair Critical-Sized Bone Defects through Enhanced Angiogenesis and Osteogenesis in Osteoporotic Rats	INT J BIOL SCI	8.2	378
6	2019	MSC exosomes alleviate temporomandibular joint osteoarthritis by attenuating inflammation and restoring matrix homeostasis	BIOMATERIALS	12.8	355
7	2019	miR-100-5p-abundant exosomes derived from infrapatellar fat pad MSCs protect articular cartilage and ameliorate gait abnormalities via inhibition of mTOR in osteoarthritis	BIOMATERIALS	12.8	352
8	2017	MSC exosome as a cell-free MSC therapy for cartilage regeneration: Implications for osteoarthritis treatment	SEMIN CELL DEV BIOL	6.2	346
9	2018	Exosomes derived from miR-92a-3p-overexpressing human mesenchymal stem cells enhance chondrogenesis and suppress cartilage degradation via targeting WNT5A	STEM CELL RES THER	7.1	319
10	2019	Desktop-stereolithography 3D printing of a radially oriented extracellular matrix/mesenchymal stem cell exosome bioink for osteochondral defect regeneration	THERANOSTICS	12.4	307

### 3.5 Journals analysis

**FIGURE 6:**
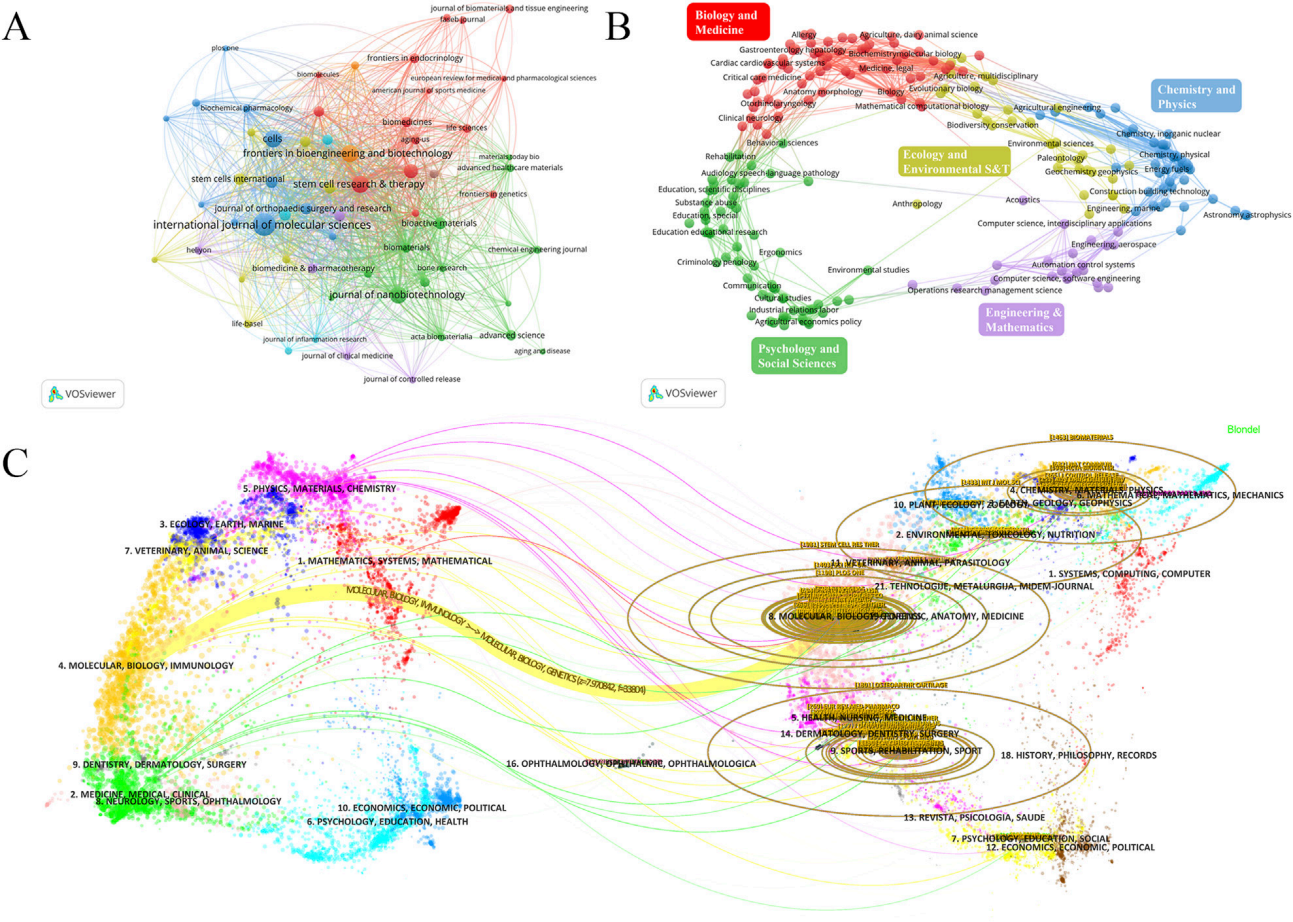
**(A)** journal network visualization. The size of the nodes represents the number of publications of the journals, and the colors represent different clusters. **(B)** Analyses of research subject areas. Different colors represent different fields. **(C)** The overlay of dual-map journals. The map is divided into two parts: the left side shows the distribution of the citing journals. The right side provides the distribution of the cited journals, which can be considered the research foundation for the former. The dots represent different journals, different colors represent the fields of interest shared by journals in the same cluster, and the ellipses represent the number of publications and the ratio of authors to publications for a journal. The length of the ellipses represents the number of authors, and the width represents the number of publications.

A total of 384 journals have published research articles on EVs in the field of DMDs. We set a minimum threshold of five articles per journal and selected 60 journals for visualization analysis (Figure 6). Table 4 lists the top 10 journals by publications, along with their number of publications (NP), citation (NC), H-index, and impact factor (IF), and most of which have grown rapidly since 2016 (Supplementary Figure 4B). The journal with the most publications in this field is INT J MOL SCI (NP = 55), the most influential journal is STEM CELL RES THER (NC = 2,303, H-Index = 23), and the top three journals by impact factor are BIOACT MATER (IF = 18), J NANOBIOTECHNOL (IF = 10.6), and STEM CELL RES THER (IF = 7.1).

**TABLE 4:** The top 10 journals ranked by publications with NP, NC, H-index, and IF.

Rank	Journal	NP	NC	H-index	If (2023)
1	INT J MOL SCI	55	756	15	4.9
2	STEM CELL RES THER	32	2,303	23	7.1
3	FRONT BIOENG BIOTECH	32	681	16	4.3
4	Cells	32	565	15	5.1
5	J NANOBIOTECHNOL	32	516	11	10.6
6	Front Cell Dev Biol	23	690	15	4.6
7	J ORTHOP SURG RES	16	317	8	2.8
8	ARTHRITIS RES THER	15	672	10	4.4
9	BIOACT MATER	15	817	12	18
10	FRONT IMMUNOL	15	347	9	5.7

The journal network and density visualizations offer insights into the journals with the most publications and the collaborative relationships between them (Figure 6A; Supplementary Figure 4A). We used VOSviewer to analyze the 384 journals in this field across different categories. Most journals publish research articles concentrated in biomedicine and chemical physics, with a particular emphasis on biomedicine (Figure 6B). Table 5 lists the top 10 fields by publications, the top three fields in terms of publication output are Cell Biology (270), Biochemistry and Molecular Biology (160), and Experimental Medicine Research (160). The number of publications across various fields began to increase rapidly in 2019, with biology-related fields dominating this growth. However, since 2021, there has been a decline in publication within biology-related fields, while other interdisciplinary fields have shown more significant growth. This trend indicates that interdisciplinary studies are becoming the future focus, rather than being solely confined to biology (Supplementary Figure 5). Current research on EVs in the field of DMDs is primarily focused on basic research and clinical translation.

**TABLE 5:** The top 10 fields ranked by publications.

Rank	Field	Record count	% Of total
1	Cell Biology	270	22.843
2	Biochemistry Molecular Biology	160	13.536
3	Experimental Medicine Research	160	13.536
4	Pharmacology Pharmacy	136	11.506
5	Cell Tissue Engineering	132	11.168
6	Engineering Biomedical	116	9.814
7	Biotechnology Applied Microbiology	110	9.306
8	Chemistry Multidisciplinary	101	8.545
9	Nanoscience Nanotechnology	100	8.46
10	Orthopedics	69	5.838

The overlay of dual-map journals effectively illustrates the interdisciplinary distribution of journals, citation patterns, and shifts in research center. We used VOSviewer and CiteSpace to visualize the overlay of dual-map journals (Figure 6C; Supplementary Figures 4C–E). The curves between the maps represent citation relationships between journals on both sides, highlighting the understanding of interdisciplinary connections in this field. Currently, interdisciplinary research on extracellular vesicles in DMDs is lacking. However, as interest grows in combining extracellular vesicles with biomaterials for treatment, interdisciplinary collaboration is likely to become a future trend in this field.

### 3.6 Authors analysis

**FIGURE 7:**
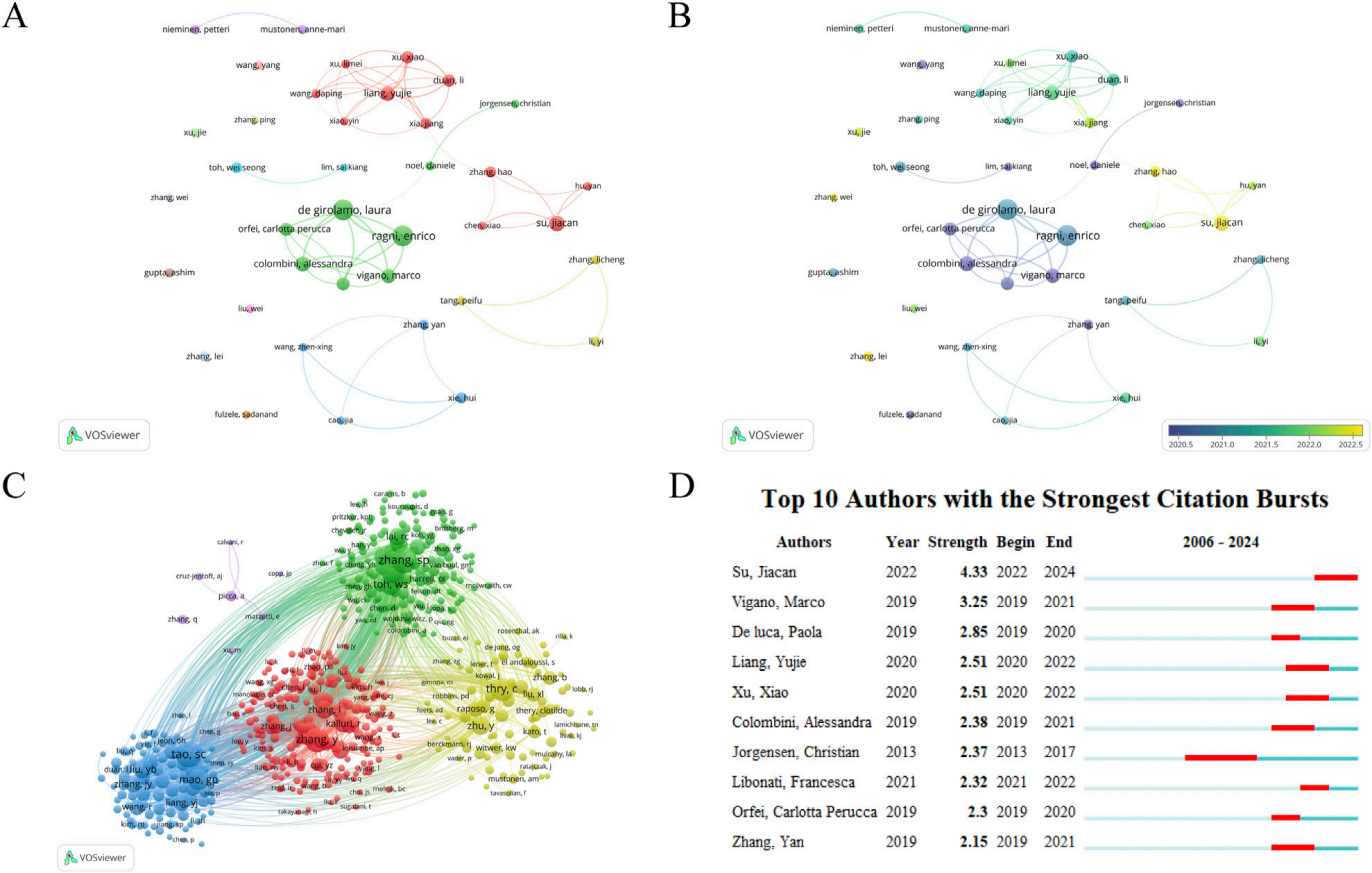
**(A)** Author network visualization. **(B)** Author overlay visualization. The size of circles in the network represents the publications of authors. The thickness of lines between circles represents the strength of collaboration among authors. The label view reflects the average publication year of authors based on the darkness of circle colors. **(C)** Author Co-citation Analysis by VOSviewer. Co-citation analysis refers to multiple authors’ articles being cited in the same literature. The size of circles reflects the citation count of authors. Lines represent co-citation relationships, and the thickness of lines can indicate the frequency of co-citation. A higher frequency of co-citation between two authors may indicate that their research areas or topics are closely related. If the two authors have different academic backgrounds, it can reflect the interdisciplinary research potential of this field. **(D)** Top 10 authors with the strongest citation bursts.

6,720 authors have published research articles on EVS in the field of DMDs. We set a minimum threshold of 7 articles per author and selected 38 authors for visualization analysis (Figure 7). Table 6 shows the top 10 authors by publications, including their affiliations, countries, publication counts, citation counts, and H-index. The top three authors are Ragni, E (23), De Girolamo, L (22), and Su, JC (16). The most cited author is Toh, WS (2066), reflecting the significance of these authors in the research field. Interestingly, six of the top 10 authors belong to the same research team at IRCCS Istituto Ortopedico Galeazzi in Italy. This team primarily focuses on the basic research and clinical translation of mesenchymal stem cells. Keeping track of their research progress provides valuable insights into the current hotspots and frontiers of this field. In Figures 7A, B, it is evident that close collaboration among authors in this field is lacking. Most collaborative relationships are confined to the same country or institution. Furthermore, there is almost no collaboration among authors from the top 10 countries, which hinders the field’s progress. Encouraging international academic exchange and collaboration is essential for its development.

**TABLE 6:** The top 10 authors ranked by publications with their affiliations, countries, NP, NC, and H-index.

Rank	Author	Affiliation	Country	NP	NC	H-index
1	Ragni, E	IRCCS Istituto Ortopedico Galeazzi	Italy	23	509	14
2	De Girolamo, L	IRCCS Istituto Ortopedico Galeazzi	Italy	22	476	13
3	Su, JC	Shanghai Jiao Tong University	China	16	791	12
4	Colombini, A	IRCCS Istituto Ortopedico Galeazzi	Italy	15	438	12
5	Orfei, CP	IRCCS Istituto Ortopedico Galeazzi	Italy	14	290	8
6	Viganò, M	IRCCS Istituto Ortopedico Galeazzi	Italy	14	434	12
7	Liang, YJ	Jining Medical University	China	13	744	9
8	De Luca, P	IRCCS Istituto Ortopedico Galeazzi	Italy	13	350	9
9	Toh, WS	National University of Singapore	Singapore	12	2066	9
10	Xie, H	Xiangya Hospital	China	12	623	9

The total number of authors involved in the author co-citation analysis is 36,373. We set a minimum threshold of 20 citations per author and selected 577 authors for visualization analysis (Figure 7C). Tao SC, Zhang SP, and Zhang Y are the top three most cited authors, exerting significant academic influence in this field, potentially providing a foundation or inspiration for future studies. Among the top 10 authors with the strongest citation bursts (Figure 7D), Su Jiacan has shown the highest burst strength in the past 2 years (4.33). Notably, the burst periods for all top 10 authors have occurred in recent years, reflecting the research hotspots of their respective periods. By analyzing these authors and their research, other researchers can gain insights into academic trends, stay updated on the latest developments, and follow emerging dynamics in the field.

### 3.7 Institutions analysis

**FIGURE 8:**
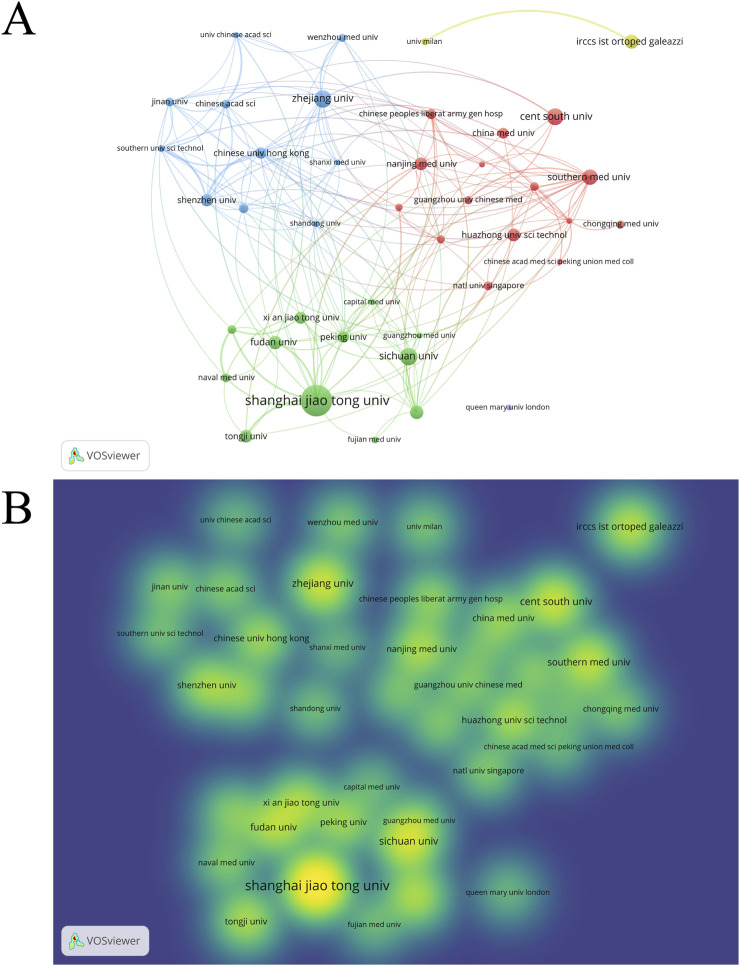
**(A)** institution network visualization. **(B)** Institution density visualization by VOSviewer.

1,452 institutions have published research articles on EVs in the field of DMDs. We set a minimum threshold of 10 articles per institution and selected 41 institutions for visualization analysis (Figure 8). Table 7 displays the top 10 institutions ranked by publications, along with the number of their publication and citation. Shanghai Jiao Tong University in China ranks first with 71 publications. Notably, 9 of the top 10 institutions are from China, highlighting the country’s high level of activity in this field. Figures 8A, B illustrate the publication output and institutional collaborations, emphasizing the lack of international cooperation, as most collaborations take place within domestic institutions. Statistical analysis revealed no significant correlation between the number of publications (NP) and total link strength among institutions (p > 0.05). Similarly, grouping institutions into high, medium, and low total link strength categories and comparing their NP revealed no statistically significant differences among the three groups (Supplementary Figures 1B, D). These findings suggest that, at the institutional level, total link strength may not have a direct influence on publication output in this field. However, this lack of statistical significance could be attributed to several factors. First, differences in institutional research capacity, resource allocation, and focus areas may moderate the relationship between collaboration intensity and NP. Second, total link strength reflects the quantity of collaborations but does not account for their quality, which may play a more critical role in driving publication output. Nevertheless, the importance of mutual collaboration in advancing research cannot be entirely dismissed. In addition, Figure 8B also uses color gradients to clearly represent the publication volume of each institution. Supplementary Figure 6 illustrates the cross-linking between countries, institutions, and authors for the top 10. It is evident that the most prominent research power is concentrated in China, the United States of America, Singapore and Italy.

**TABLE 7:** The top 10 institutions ranked by publications with their NP and NC.

Rank	Institutions	NP	NC
1	Shanghai Jiao Tong University	71	2657
2	Central South University	37	1391
3	Sichuan University	34	572
4	Zhejiang University	34	1275
5	Southern Medical University	30	359
6	IRCCS Istituto Ortopedico Galeazzi	28	600
7	Peking University	26	592
8	Fudan University	25	954
9	Sun Yat Sen University	25	1135
10	Nanjing Medical University	24	1017

## 4 Discussion

### 4.1 General information

In this study, we conducted a bibliometric analysis of literatures on EVs research in DMDs from 2006 to 2024. The aim is to comprehensively understand the current research status and trends in this field, providing scholars with a systematic reference framework to understand important achievements and frontiers. Our results indicate that the period from 2006 to 2016 was the initial stage of research in this field, with annual publications fewer than 10 articles. However, since 2016, the field has experienced explosive growth, with annual publications exceeding 100 in 2020. Notably, in the past 2 years alone, the publication volume doubled in 2022. Up to now, the continuous rapid growth in annual publication volume indicates that EVs research in DMDs is gaining increasing attention and research enthusiasm.

The visualization analysis of countries, institutions, and authors shows that China, the United States, and Italy are the top three countries in terms of publication volume in this field. China leads significantly, with 716 publications (NP) and 19,739 citations (NC), far surpassing other countries. However, China’s collaboration with other countries is relatively low (link strength = 94), with most research being independently conducted. In contrast, the United States has only 132 publications, but its link strength is as high as 111, indicating a strong emphasis on collaborative research with other countries. This model of international cooperation and extensive academic exchange is essential for the faster and better development of this field. The top three institutions by publication volume are Shanghai Jiao Tong University (71), Central South University (37), and Sichuan University (34). Understanding their publication volume, citation counts, and research directions provides valuable insights for choosing research collaborations and academic exchanges.

Ragni, E, De Girolamo, L, and Su, JC are currently the top three authors by NP and have conducted extensive research in this field. Ragni, E and De Girolamo, L are from the same laboratory, focusing mainly on the roles of secreted factors and extracellular vesicles derived from mesenchymal stem cells in regenerative medicine. This includes research related to DMDs, particularly the therapeutic mechanisms of mesenchymal stem cells from different sources for diseases such as OA (Ragni et al., 2019; Ragni et al., 2020). Su, JC mainly focuses on the basic research and clinical translation of engineered extracellular vesicles for treating degenerative diseases. This includes using bone-targeting extracellular vesicles to deliver therapeutic drugs for treating OP and other degenerative diseases, as well as research and applications related to biomaterials (Hu et al., 2021; Jiang et al., 2022; Liu et al., 2022a; Liu et al., 2022b).

We analyzed journals that published articles on EVs related to DMDs. The top three journals by NP are INT J MOL SCI (IF = 4.9, Q1), FRONT BIOENG BIOTECH (IF = 4.3, Q1), and STEM CELL RES THER (IF = 7.1, Q1). Among the top 10 journals by NP in this field, nine are Q1 journals, including BIOACT MATER, the top journal in the biomaterials field. This indicates that the field attracts many excellent scholars and the quality of published research is high. The overlay map reveals that the main fields of EVs research in DMDs are biomedicine and chemical physics. Combining this with the dual-map overlay results, it reveals a lack of multidisciplinary integration in this research direction. The focus is mainly on molecular biology, biology, and immunology, with a strong emphasis on basic research and a noticeable shortage of clinical studies.

The keyword analysis highlights the main research directions and hotspots in the field. Additionally, the time zone map provides insights into the progression of research over time. The red cluster relates to how EVs regulate signaling pathways, immune responses, and gene expression, impacting bone and muscle metabolism, regeneration, and degeneration. This cluster addresses pathological processes linked to aging, osteoporosis, and other DMDs. The green cluster focuses on how EVs regulate immune cells (such as T cells and macrophages) and their secretions to promote cartilage and bone repair, as well as their combination with stem cells, regenerative medicine, and biomaterials to advance treatment strategies. The blue cluster highlights the role of EVs in diagnosing and treating DMDs by promoting bone and cartilage regeneration, enabling targeted drug delivery, and integrating with biomaterials. The purple cluster explores the regulation of cartilage and bone degeneration, inflammatory responses, and the cellular microenvironment, influencing disease progression. Keywords in the yellow cluster mainly focus on the role of EVs in tissue repair, osteogenesis and chondrogenesis, regulation of inflammatory responses, and relevant biomarkers. The connections between the red, green, and blue clusters indicate substantial overlap among these research directions (Figures 4A, B).

Moreover, the research history of extracellular vesicles shows a progression from initially studying exosomes, to extracellular vesicles, and more recently, to small extracellular vesicles. Exosomes are named based on their biogenesis, originating from the endosomal system. When the subcellular origin of vesicles cannot be determined, the term “exosomes” is not recommended for naming extracellular vesicles. Subsequently, methods such as differential ultracentrifugation (dUC) have been employed to differentiate extracellular vesicle subtypes by size, with those smaller than 200 nm classified as small EVs (sEVs) (Crescitelli et al., 2021). Additionally, study has identified non-vesicular extracellular particles, which can co-isolate with extracellular vesicles, as multimolecular assemblies lacking a lipid bilayer, and their influence on extracellular vesicle studies is increasingly recognized. In summary, the evolving terminology of extracellular vesicles reflects the advancements in this field (Welsh et al., 2024). Nodes with purple outer rings (e.g., exosomes, articular cartilage, extracellular vesicles, mesenchymal stem cells) indicate keywords with high betweenness centrality, playing crucial bridging roles in this field. Additionally, by examining the newly emerged keywords in the past 2 years, we can identify the research frontiers (Figure 4C). Based on keywords emerging in the past 2 years, the research hotspots of EVs in the field of DMDs primarily focus on the following aspects: First, the application of EVs in bone metabolism and bone joint diseases, especially in osteoarthritis, senile osteoporosis, as well as bone reconstruction and repair, has become a key area of research. Additionally, engineered exosomes have gained widespread attention for enhancing therapeutic effects, particularly in bone regeneration and joint repair. Studies have also delved into the characteristics and potential applications of different types and sources of EVs, such as exosomes, apoptotic vesicles, nanovesicles, and plant-derived vesicles, in bone joint diseases. In terms of signaling pathways, the role of the PI3K/Akt/mTOR pathway in bone metabolism mediated by EVs has been highlighted, while the mechanisms by which EVs regulate cellular responses through receptor interactions are also becoming clearer. Stem cell-derived EVs, especially those derived from adipose mesenchymal stem cells and mesenchymal stem cells, are considered an important research direction for tissue repair and regeneration. Furthermore, the potential of EVs in alleviating symptoms such as chronic pain, synovitis, and stress responses has also attracted attention. As these fields continue to advance, EVs are showing increasing promise for application in the treatment of DMDs.

In the co-citation analysis, the top 10 cited articles each have over 200 citations, reflecting the foundation of this research field and the main research directions over various periods, indicating high influence. With the most-cited being “MSC exosomes mediate cartilage repair by enhancing proliferation, attenuating apoptosis and modulating immune reactivity” by Zhang S et al., published in BIOMATERIALS in 2018, with 607 citations. The second and third most-cited articles are “Exosomes derived from miR-140-5p-overexpressing human synovial mesenchymal stem cells enhance cartilage tissue regeneration and prevent OA of the knee in a rat model” (514 citations) and “Exosomes derived from human embryonic mesenchymal stem cells promote osteochondral regeneration” (482 citations). In 2014, Kato T et al. revealed the role of EVs in the pathogenesis of OA (Kato et al., 2014). And then, Zhang S (2016), and Tao SC (2017) et al. proposed the limitations of MSC-based cell therapies for cartilage repair and discovered the potential of cell-free therapies, opening new avenues for the treatment of DMDs (Zhang et al., 2016; Tao et al., 2017), which might be the reason for the rapid increase in the annual publications in this research field since 2016. Subsequently, Zhang S (2018) et al. addressed the unresolved issues in these two articles and revealed the mechanism by which extracellular vesicles derived from mesenchymal stem cells mediate cartilage repair, including promoting chondrocyte proliferation, migration, and matrix formation, inhibiting chondrocyte apoptosis, and modulating immune reactivity, laying the foundation for subsequent research (Zhang et al., 2018). Due to the limitations of cell therapy such as tumorigenicity and biocompatibility (Tan et al., 2024), safer and more effective cell-free therapies are emerging. This article provides an important theoretical basis for subsequent research on cell-free therapies for the treatment of diseases such as OA. This might also be the reason for its high citation.

### 4.2 Frontiers and hotspots

Keywords can reflect the research hotspots and development trends in this field. We analyze keyword co-occurrence, clustering, keyword bursts, and the timezone view to understand the research hotspots and frontiers in this field.

#### 4.2.1 The role of EVs in the treatment of DMDs

Currently, the clinical treatment of DMDs mainly relies on drug interventions to alleviate symptoms (LeBoff et al., 2022; Reid and Billington, 2022), making it difficult to achieve complete recovery. For severe cases, such as patients with advanced osteoarthritis (OA) where conservative treatments are ineffective or pain significantly impacts their quality of life, total joint replacement surgery may be necessary. However, this procedure often brings substantial physical pain and financial burden to patients (Abramoff and Caldera, 2020). With the continuous progress of related research, stem cell therapy has become a focal point in the field of regenerative medicine. Because of their rapid proliferation, strong differentiation potential, and ability to migrate to injury sites (Margiana et al., 2022), mesenchymal stem cells (MSCs) from both autologous and allogeneic sources have been extensively researched for treating various DMDs, demonstrating promising therapeutic outcomes (Harrell et al., 2019). However, there are also limitations and risks, such as heterogeneity of MSCs from different sources, immune rejection, and tumorigenicity, which restrict their clinical application (Musiał-Wysocka et al., 2019). Recent research has revealed that the function of MSCs in tissue regeneration is primarily driven by their paracrine effects. EVs in the secretome have attracted significant attention due to their benefits, including low immunogenicity, targeted delivery, and biocompatibility (Tang et al., 2021). Growing evidence suggests that cell-free therapy using EVs has emerged as a highly potential treatment approach for DMDs.

EVs can act as mediators of crosstalk between multiple organs. Extracellular vesicles secreted by other organs (such as muscle and liver) influence bone metabolism through circulation, thereby contributing to the treatment of degenerative diseases. The study by Ma S et al. showed that mu-EVs promote glycolysis in bone marrow mesenchymal stem cells (BMSCs) by delivering lactate dehydrogenase A to BMSCs, which in turn promotes the osteogenic differentiation of BMSCs. This offers a new approach for the treatment of OP and also reveals a new mechanism by which skeletal muscle affects bone metabolism (Ma et al., 2023). Additionally, research by Lin L and colleagues has found an interaction between liver and bone metabolism. Liver-specific SIRT2 deficiency can ameliorate bone loss caused by OP. Extracellular vesicles from SIRT2 ^(−/−)^ hepatocytes, carrying LRG1, are transferred to bone marrow-derived macrophages (BMDM) via circulation and inhibit osteoclastogenesis by suppressing the activation of NF-κB p65, thereby serving a therapeutic role in OP (Lin et al., 2023).

Engineered exosomes target disease-related receptor cells to deliver therapeutic cargo, achieving precise treatment. Natural EVs have low drug loading capacity and lack the ability to be effectively delivered to target sites for clinical applications. Some engineering methods can modify the surface of EVs to deliver them to specific target tissues and further explore methods for loading therapeutic drugs into EVs, thereby achieving efficient drug delivery (Komuro et al., 2022). Zhang H et al. engineered a cartilage affinity peptide (CAP) on the EVs surface through lipid insertion, obtaining chondrocyte-targeting exosomes—CAP-Exo. Then, they loaded siRNA targeting MMP13 (siMMP13) into these exosomes, resulting in CAP-Exo/siMMP13. These exosomes delivered siRNA to chondrocytes, specifically silencing MMP13 in chondrocytes, thereby alleviating cartilage degradation caused by OA (Zhang et al., 2024). Similarly, Yan W et al. used an engineered method where sortase A was used to link EVs and cartilage affinity peptide (CAP), followed by incubation with cholesterol-modified ASO-MMP13 to construct chondrocyte-targeting drug delivery exosomes (CAP-exoASO), which also reduced cartilage damage caused by OA (Yan et al., 2024). Besides single-surface engineering of EVs, more efficient drug loading can also be achieved through combined engineering approaches. Liu W et al. utilized dual-engineered EVs by loading exogenous miR-223 into hUC-EVs via electroporation and engineering a collagen II-targeting peptide (WYRGRL) onto the surface of hUC-EVs, thereby enabling more specific and effective RNA delivery to chondrocytes, which provides a more effective treatment for OA (Liu W. et al., 2023). Moreover, advanced exosome preparation methods can greatly increase the success rate of clinical translation by improving the yield of exosomes used for disease treatment. Yang Z et al. invented a revolutionary exosome preparation method called cell nanoporation, which combines the steps of purification and drug loading. By using nanoporation, cells secrete exosomes loaded with target mRNA, significantly improving their yield, targeting, and applicability (Yang et al., 2020).

The therapeutic efficacy of EVs relies on their internalization by target cells and/or their close interaction with receptors on the target cell surface, both of which necessitate the prolonged presence of EVs at the target site. Therefore, although EVs have therapeutic potential, their effects are short-lived when used alone. EVs administered systemically have a short half-life and are rapidly eliminated from the body, making it challenging to attain sustained therapeutic effects with EVs treatment (Leung et al., 2022). Studies have shown that biomaterial-based delivery systems can extend the retention time of EVs at target sites. Additionally, local delivery based on biomaterials can reduce the dose of EVs required to achieve therapeutic effects, thereby reducing associated costs and potential side effects (Murali and Holmes, 2021). Therefore, the combination of EVs and biomaterial-based delivery modes is gaining increasing attention. Chen M et al. used the CRISPR/Cas9 method to create cartilage-affinity peptide (CAP)-conjugated exosomes containing FGF18-targeted gene editing tools (CAP/FGF18-hyEXO), efficiently stimulating the FGF18 gene in OA chondrocytes at the genetic level *in vivo*. In addition, CAP/FGF18-hyEXO is encapsulated in methacrylic anhydride-modified hyaluronic acid (HAMA) hydrogel microspheres via microfluidics and photopolymerization, forming an injectable microgel system with a renewable hydration layer (CAP/FGF18-hyEXO@HMs). This system provides continuous lubrication to counter friction and wear, demonstrating the ability to work synergistically to promote cartilage regeneration, reduce inflammation, and prevent ECM degradation, both *in vitro* and *in vivo* (Chen M. et al., 2024). Pang L, Wan J, and Li X utilized methacryloyl gelatin (GelMA) hydrogels to load engineered exosomes for therapeutic purposes, allowing for sustained release at the treatment site and addressing the challenges of rapid elimination and limited retention of exosomes. In the study by Pang L et al., GelMA hydrogels loaded with MSC-NVs (GelMA-NVs) regulated chondrogenesis and macrophage polarization through MSC-NVs, providing a therapeutic effect on OA (Pang et al., 2023). Wan J et al. used GelMA hydrogels to load engineered exosomes modified with targeting peptides, enhancing the targeting and retention capabilities of exosomes, thereby maximizing their therapeutic effects. In addition to enhancing the retention of exosomes, biomaterials can also be used to achieve controlled release strategies for exosomes, enabling more precise treatments (Wan et al., 2023; Li X. et al., 2024). Li S et al. proposed a hydrogel composite with a logic gate function, the KM13E@PGE system. It uses matrix metalloproteinase 13 (MMP13)-sensitive self-assembling peptide hydrogel (KM13E) and polyethylene glycol diacrylate/gelatin methacryloyl (PGE/GelMA) hydrogel microspheres to encapsulate interleukin-10 (IL-10) EVs, which promote M2 macrophage polarization, and SRY-box transcription factor 9 (SOX9) EVs, which enhance cartilage matrix synthesis. The release times of the two types of EVs are controlled to achieve an initial anti-inflammatory effect followed by cartilage repair (Li S. et al., 2024). This is an effect that cannot be achieved by a single extracellular vesicle. Developing biomaterials that address specific therapeutic needs is crucial for the clinical translation of natural and engineered exosomes and aligns with the trend of interdisciplinary research, which has seen increasing interest in recent years.

Different pretreatments and culture conditions can influence the secretion of exosomes by altering the microenvironment. Studies have shown that controlling stem cell growth conditions through medium pretreatment with physical properties (such as culture systems and cell density), oxygen tension, and soluble factors can directly impact paracrine activity by modulating the cargo and biogenesis of EVs (Mas-Bargues and Borrás, 2021), thus improving therapeutic effectiveness. Hu H et al. discovered that hypoxic pretreatment enhances the biological functions of mesenchymal stem cells in challenging microenvironments and stimulates the production of small EVs with beneficial therapeutic properties. The produced sEVs (HP-sEVs) can reduce the inflammatory microenvironment of intervertebral disc degeneration, stimulate the proliferation of nucleus pulposus (NP) cells, and enhance proteoglycan synthesis and collagen production, providing a new therapeutic strategy for treating IVD degeneration (Hu et al., 2023). Interestingly, It has been shown that EVs from aging mesenchymal stromal cells have a significantly impaired protective effect against OA. They also induce catabolic metabolism and pro-inflammatory gene expression, demonstrating that cellular aging is a factor that affects the secretion of EVs, thereby altering their original biological functions (Jérémy et al., 2024).

Besides human-derived EVs, studies have shown that plants can also secrete exosome-like nanovesicles containing miRNA, bioactive lipids, mRNA, and proteins, which can exert regenerative activity and have therapeutic effects on diseases such as OP. Hwang JH et al. showed that exosome-like nanovesicles from yam (YNV) can activate the BMP-2/p-p38-dependent Runx2 pathway to stimulate osteogenesis, providing a therapeutic effect on OP (Hwang et al., 2023). Zhan W et al. found that puerarin-derived exosome-like nanovesicles (PELNs) promote autophagy by degrading the gut microbiota metabolite trimethylamine-N-oxide (TMAO), which in turn promotes the differentiation and mineralization of hBMSCs, showing potential as a treatment for OP (Zhan et al., 2023).

#### 4.2.2 EVs as biomarkers for the diagnosis of DMDs

Although new treatment methods for various DMDs have emerged in recent years, clinical translation still requires some time, and safety and efficacy need further verification through more clinical trials. Currently, conservative treatment remains the primary clinical approach, making timely early diagnosis and intervention particularly important. As early as 2004, EVs demonstrated great potential as disease detection tools. In the following years, the research interest in EVs as biomarkers for various diseases (Das et al., 2024) [such as cancer (Kumar M. A. et al., 2024; Yu et al., 2022; Lee et al., 2023), neurological disease (Chatterjee et al., 2024; Ricklefs et al., 2024; Kumar A. et al., 2024), metabolic diseases (Barreiro et al., 2020; Camino et al., 2022), etc.] has continuously increased. In 2016, ExoDx™ Lung (ALK) became the first biomarker to successfully complete clinical trials, marking a milestone for EVs as disease diagnostic biomarkers by diagnosing lung cancer through the detection of EMLA-ALK mutations (Sheridan, 2016). In the field of DMDs, there is substantial research on EVs as biomarkers for diagnosing OA. This research primarily involves early diagnosis of OA, identification of inflammation types, susceptibility assessment, and differential diagnosis of various types of joint diseases, enabling precise and personalized treatment of OA (Liu Z. et al., 2023).

Furthermore, existing studies have indicated that miRNAs from EVs can serve as potential biomarkers for early diagnosis of OP and prediction of fragility fracture risk (Huber et al., 2023; Pepe et al., 2022). Currently, there is a shortage of studies focusing on other degenerative diseases such as sarcopenia. While the prospect of EVs as disease diagnostic biomarkers is exciting, their limitations in specificity, reproducibility, and lack of single/unified molecules related to early disease processes, along with a lack of clinical studies in this field, require further research to be addressed.

#### 4.2.3 The role of EVs in the mechanisms of DMDs development

As an essential medium for intercellular communication, EVs can transport therapeutic contents, and EVs derived from pathogenic cells can mediate disease occurrence by transporting pathogenic contents. Aging is a major cause of degenerative diseases, so EVs derived from various aging cells or those carrying aging-regulating factors may be involved in the disease development process. The reduced expression of miR-494-3p in exosomes from aging osteocytes suppresses osteogenic differentiation and promotes age-related bone loss via the PTEN/PI3K/AKT pathway (Yao et al., 2024). There have been previous reports that infrapatellar fat pad (IPFP) is closely related to the occurrence and progression of knee OA. Cao Y et al. found that small EVs (sEVs) secreted by the IPFP of OA patients can be delivered to articular chondrocytes. The high levels of let-7b-5p and let-7c-5p within these sEVs can aggravate the progression of OA by directly reducing the expression of the senescence-negative regulator Lamin B receptor (LBR) (Cao et al., 2024). M1 macrophages can impair joint homeostasis and lead to OA progression by producing nitric oxide and pro-inflammatory cytokines. Recently, studies have first demonstrated that EVs secreted by inflammatory macrophages induce atypical pyroptosis of chondrocytes, leading to cartilage catabolism and OA development (Ebata et al., 2023). Additionally, Liu B et al. found that the internalization of exosomes derived from inflammatory fibroblast-like synoviocytes (inf-exo) enhances M1 polarization of macrophages by triggering glycolysis activation, further inducing OA-like phenotypes in co-cultured chondrocytes. *In vivo* experiments also confirmed that inf-exo induces synovitis and exacerbates OA progression (Liu et al., 2024). In-depth research into the mechanisms by which EVs mediate disease occurrence can provide new targets and strategies for disease treatment. For example, limiting the uptake of EVs by receptor cells can inhibit their pathogenic effects. The latest research by Lin S et al. found that exosome-like nanovesicles derived from osteosarcoma, even after content removal, still possess targeting ability. By competitively inhibiting the uptake of normal osteosarcoma-derived exosomes by lung fibroblasts, they can inhibit lung fibroblast activation, pre-metastatic niche formation, and osteosarcoma lung metastasis (Lin et al., 2024). This research can also provide a reference for treating DMDs.

### 4.3 Limitations and future perspectives

EVs are gaining increasing attention in the field of DMDs research due to their roles in treatment, diagnosis, and elucidation of disease mechanisms. Currently, research in this field mainly focuses on the role of EVs in the treatment of DMDs and on using EVs to investigate new disease mechanisms, providing new therapeutic targets, essentially serving disease treatment. There are currently several limitations to using EVs for the treatment of DMDs: 1) The efficiency of EVs isolation and purification is not high enough, making it difficult to achieve satisfactory purity while maintaining high isolation efficiency. There is a lack of standardized strategies for isolation, purification, and storage, leading to poor reproducibility in experiments. 2) Current understanding of EVs biogenesis, internalization, and cargo transport processes is very limited. 3) Although engineering EVs can improve therapeutic effects and ability to target bone tissue, these strategies bring unpredictable immune responses and elevated production costs. Modifying the surface of EVs may cause the immune system to recognize them as foreign objects, triggering host immune responses, leading to clearance or reduced efficacy. These modifications may also induce toxicity or adverse reactions, posing potential risks to the body. 4) A more specific understanding of the mechanisms by which EVs mediate disease occurrence is needed to enable precise intervention by inhibiting the formation, release, or uptake of relevant EVs. 5) There is limited understanding of the mechanisms behind the heterogeneity of EVs, the disruption caused by non-vesicular extracellular nanoparticles on EVs research, and the functional discrepancies among different EVs subgroups. Identifying which EVs subgroups are functionally active or contain specific therapeutic cargoes is essential for enhancing their effectiveness and increasing repeatability. Most of these problems are common to EVs in the field of regenerative medicine, and specific to DMDs, for example, most of the studies often neglect the effects of different pathological changes at different times of OA on the localization and biodistribution of EVs, the distribution of EVs used for treatment not in cartilage are unclear. The dosage level of EVs for OA treatment is unclear. The different methods of OA model construction in preclinical studies have different effects on EVs. And the therapeutic effects proposed in preclinical studies lack the clinical trials to test. These can affect the final therapeutic outcome and the efficiency of clinical translation. Moreover, most of the current attention to EVs for the treatment of OA is focused on the articular cartilage (Karoichan et al., 2024). But OA is a multifactorial disease, the other causative factors and Pain during disease progression should be considered at the same time. In future studies, simultaneous intervention of all aspects through various modification methods and biomaterials should be attempted, which may lead to better therapeutic outcomes. Other DMDs have the similar problems. The great potential of EVs for the early diagnosis of diseases has been widely recognized. Because of the absence of effective therapeutic tools currently, early diagnosis of degenerative musculoskeletal diseases is particularly important. However, there are relatively few studies in this field, which is an important research direction in the future.

In future research within this field, it is essential to avoid homogenization and focus on addressing the most pressing issues and challenges. To overcome the current limitations of EVs in DMDs treatment, future research should focus on several key areas: 1) Efforts should be made to develop standardized protocols for the isolation, purification, and storage of EVs. This will ensure high purity, reproducibility, and efficiency in experiments. Advanced techniques, such as optimized ultracentrifugation or immunoaffinity-based methods, should be explored for scalable and reliable EV isolation. 2) Future studies should prioritize investigating the biogenesis of EVs, their internalization by target cells, and the mechanisms of cargo loading and transport. This will help to unveil the fundamental processes that dictate EVs’ function and potential therapeutic roles. 3) Engineering EVs holds great promise for enhancing therapeutic activity, but the associated immune responses and high production costs need to be addressed. Future research should focus on optimizing surface modifications to reduce immune recognition, while simultaneously developing more cost-effective production methods such as large-scale cell culture systems. 4) There is a need for more precise interventions targeting the specific mechanisms by which EVs mediate disease onset and progression. This could involve developing strategies to regulate EV formation, release, and uptake, enabling targeted therapies that are more effective in treating DMDs. 5) The functional discrepancies among different EV subgroups must be further explored to identify which subsets are functionally active or carry therapeutic cargo. Advanced techniques such as single-particle tracking and high-throughput sequencing could help identify the specific EV populations most relevant for therapeutic applications. 6) The previous points are common issues in EVs-related research. Specifically, in DMDs, there is a need for a more detailed understanding of how different pathological conditions (such as OA) influence the localization, biodistribution, and therapeutic effects of EVs. Research should address the effects of different OA stages on EV distribution and investigate the optimal dosage levels for EV-based treatments. 7) The transition from preclinical studies to clinical trials is crucial for the success of EV-based therapies. Future research should bridge this gap by incorporating more clinical trial data, and integrating disciplines such as biomaterials science, tissue engineering, and molecular medicine to enhance the clinical applicability of EVs in DMDs treatment. 8) Lastly, enhancing international collaboration and interdisciplinary research will accelerate the development of EV-based therapies. By leveraging expertise across various fields, including regenerative medicine, nanotechnology, and bioinformatics, the clinical translation of EV-based therapies can be achieved more efficiently, ultimately benefiting patients worldwide. These strategies aim to tackle the key challenges in EV research, improving their therapeutic potential and accelerating their clinical application in DMDs.

Although bibliometric analysis offers unique and valuable insights, it is not without limitations. First, the selection of databases, article types, and language restrictions can introduce biases, potentially affecting the comprehensiveness of the results. Second, visualization tools such as CiteSpace and VOSviewer may extract data differently from the original WoS database, resulting in potential discrepancies. Furthermore, differences in their underlying algorithms can lead to variations in analysis outcomes, causing inconsistencies in the visualization and interpretation of bibliometric data. Third, while bibliometric analysis is highly effective for quantitative evaluation, it is less adept at addressing qualitative aspects, such as identifying research hotspots and emerging frontiers, which often require complementary secondary analyses. To ensure clarity in the visualization results, the number of authors and institutions was reduced by raising the threshold in the analysis. However, this approach may have a potential drawback, as it could overlook some authors and institutions with innovative research emerging in the past 2 years. Fourth, to ensure the clarity of the visualization results, the number of authors and institutions was reduced by raising the threshold in the analysis. However, this approach has a potential drawback, as it may overlook some authors and institutions with innovative research emerging in the past 2 years. Finally, bibliometric analysis relies on static snapshots of data, limiting its ability to accurately predict future research trends.

## 5 Conclusion

EVs have shown great research prospects and popularity in the field of DMDs. Since 2016, the number of publications in this field has surged, with a trend expected to continue, indicating growing interest and ongoing increase in research activity. China has the highest publication output, but less frequent collaboration with other countries compared to the United States, indicating high interest in this field within China and a need to strengthen cooperation with other countries. In addition, keyword and citation analysis reveals that biomaterial-based EVs and engineered EVs as drug delivery vehicles for treating DMDs are currently the main focus areas. However, many unresolved issues and challenges still hinder the clinical translation of basic research, which remains a major goal for future studies. Overall, this is a young but promising field of research.

## Data Availability

The original contributions presented in the study are included in the article/Supplementary Material, further inquiries can be directed to the corresponding authors.
