# Supplementary material for: A bibliometric and visualized analysis of extracellular vesicles in degenerative musculoskeletal diseases (from 2006 to 2024)
Source: Front Pharmacol. 2025 Mar 13;16:1550208. doi: 10.3389/fphar.2025.1550208 (PMC11966045; doi:10.3389/fphar.2025.1550208)
Supplement: Supplementary file 1 [file DataSheet1.docx]

Supplementary Material

## Supplementary Figures


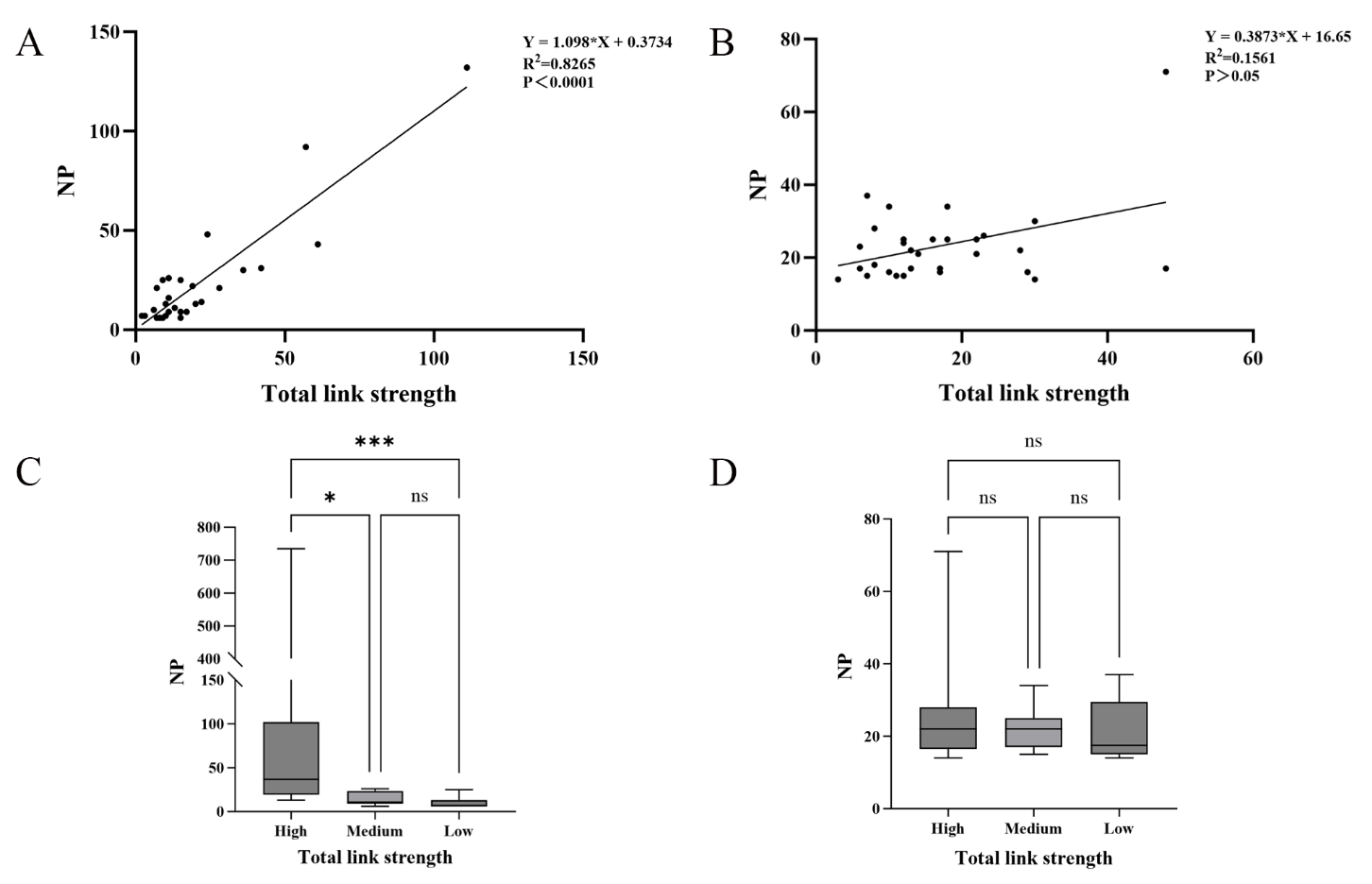


**Supplementary Figure 1.** (A) Correlation between total link strength and the number of publications (NP) for 29 countries, excluding China as an outlier due to its disproportionately high publication output. The scatter plot illustrates the statistically significant linear relationship between total link strength and NP (R^2^=0.8265, p＜0.0001). (B) Correlation between total link strength and the number of publications (NP) for 30 institutions. The scatter plot shows the linear relationship between total link strength and NP, though no statistically significant correlation was observed (R^2^=0.1561, p＞0.05). (C, D) Comparison of the number of publications (NP) across countries and institutions categorized into high, medium, and low total link strength groups using tertile classification (high: top 33%, medium: 33%-66%, low: bottom 33%). Box plots demonstrate the differences in NP among the three groups, highlighting trends in publication activity related to collaboration intensity.

**
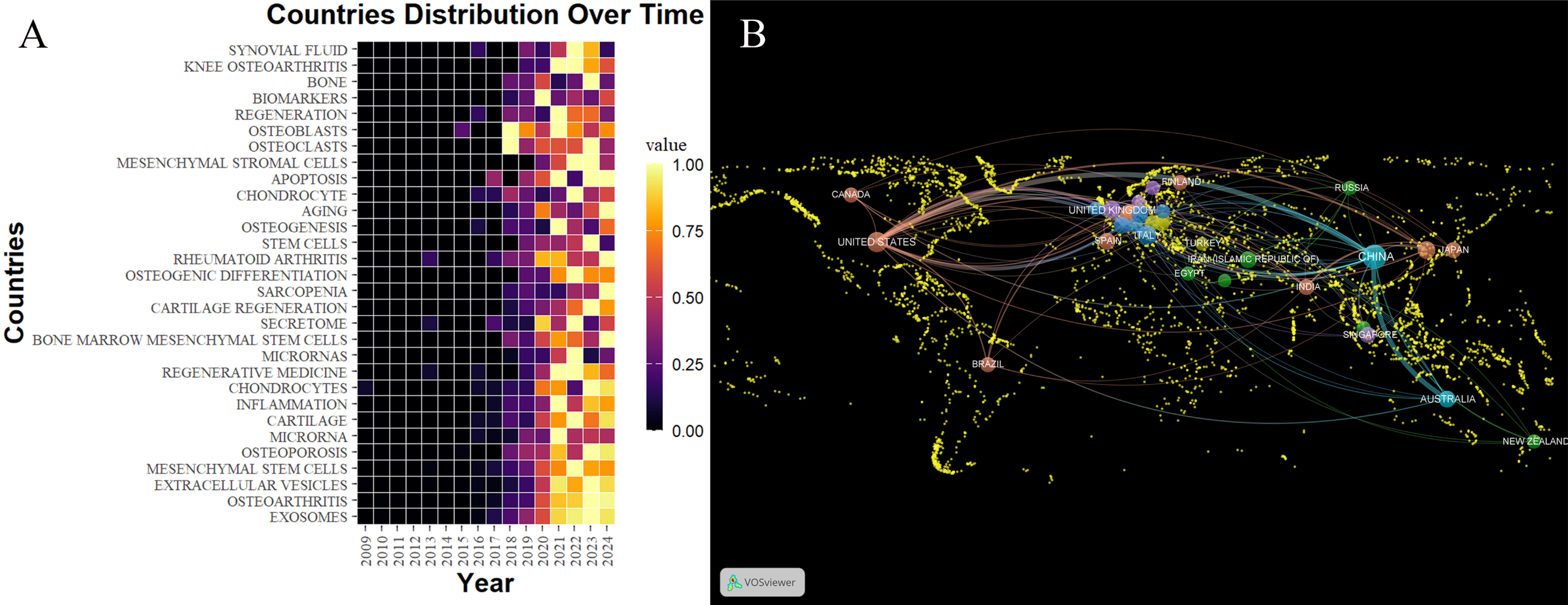
**

**Supplementary Figure 2.** (A)The heatmap of country publication over time. (B) The country collaboration map. the size of node represents the publications of each country, and the thickness of the line represents the cooperation strength between countries.

**
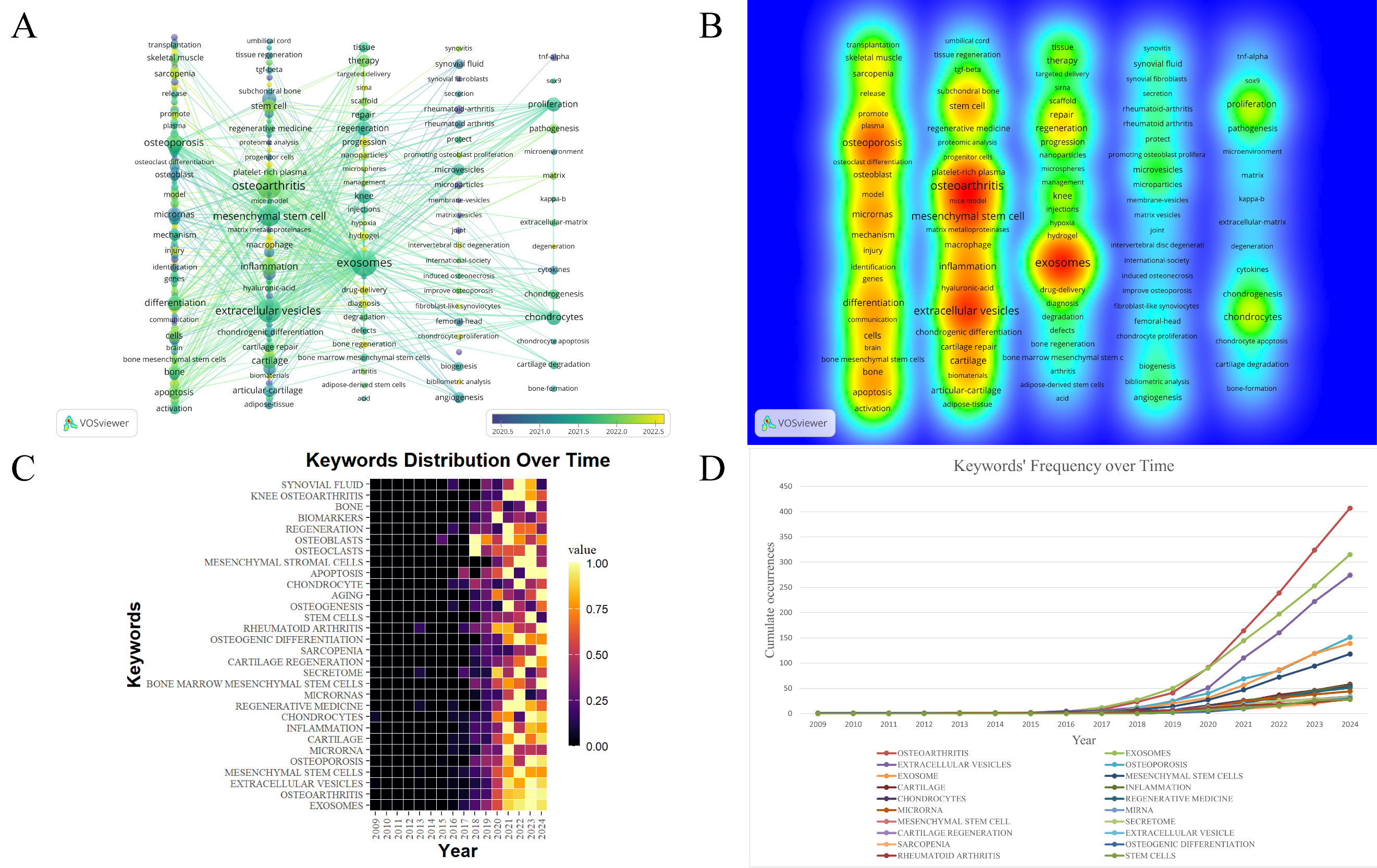
**

**Supplementary Figure 3.** (A) Keyword overlay visualization by VOSviewer. The closer the node color is to yellow, the more recent the keyword has appeared in the past two years, the closer the node color is to blue, the earlier the keyword appeared. (B) Keyword density visualization by VOSviewer. The deeper the color, the more frequently the keyword appears in the documents of this field, representing research hotspots or main areas. The annual ring positions of different keywords represent their association degree, being very close indicates that they often appear together in research. (C) The heatmap of keywords distribution over time. (D) The top 20 Keywords’ Frequency over Time.


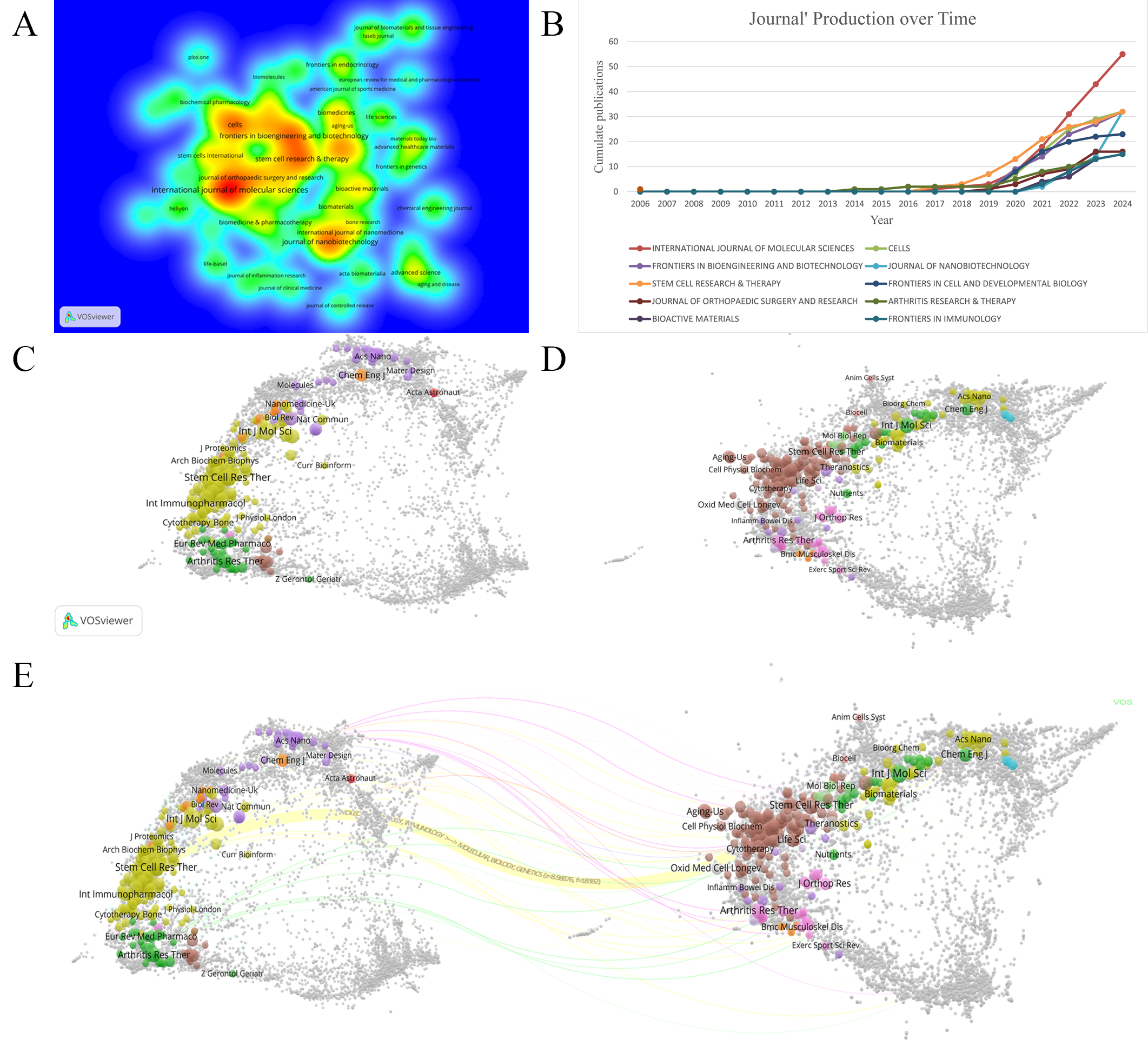


**Supplementary Figure 4.** (A) Journal network visualization. (B) The Top 10 Journal’ Production over Time. (C) Overlay Map of Citing Journals. (D) Overlay Map of Cited Journals. (E) An integrated visualization map incorporating dual-map overlays of journals generated by CiteSpace (below) and VOSviewer (above).


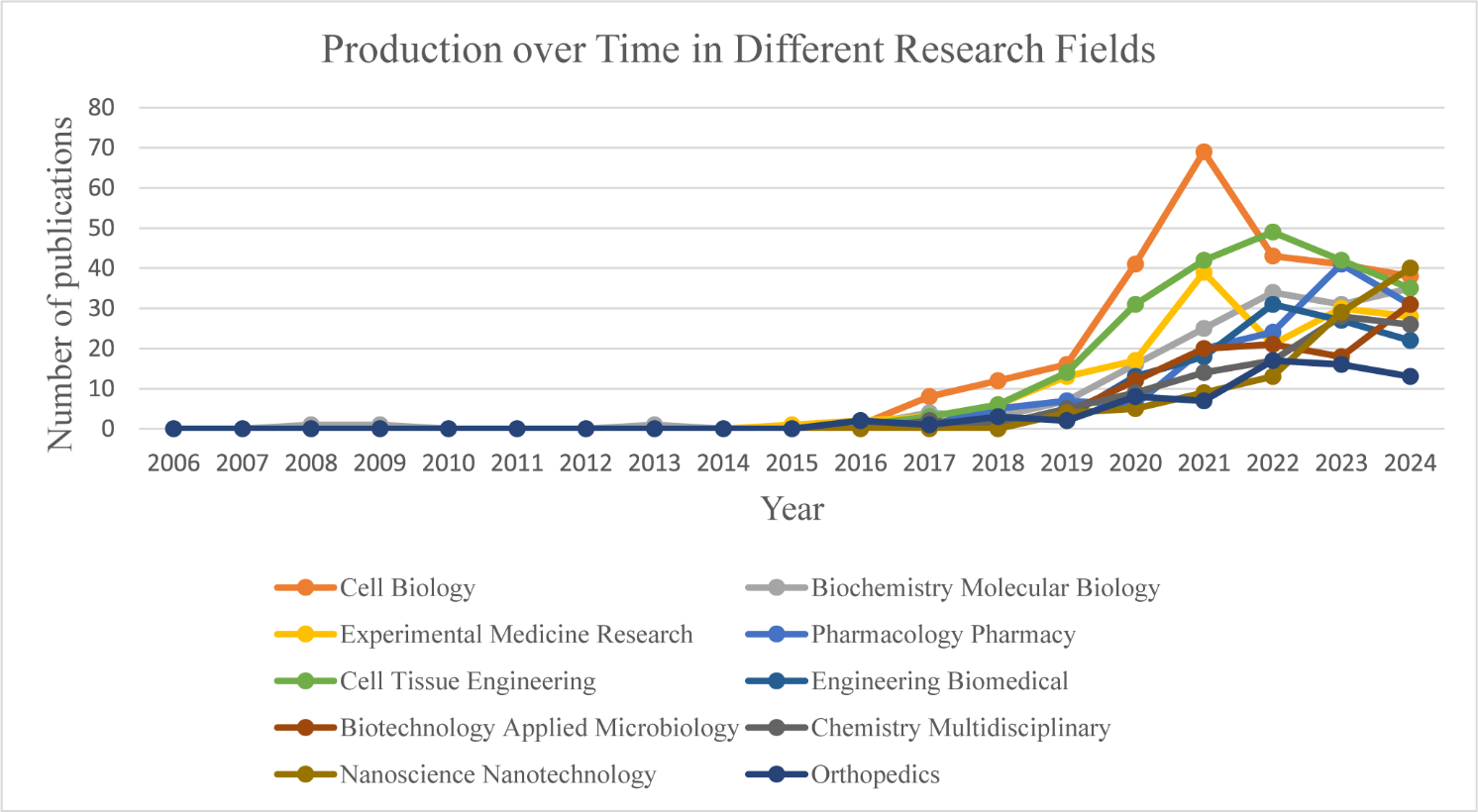


**Supplementary Figure 5.** Trends in the number of publications over time in the top 10 research fields.


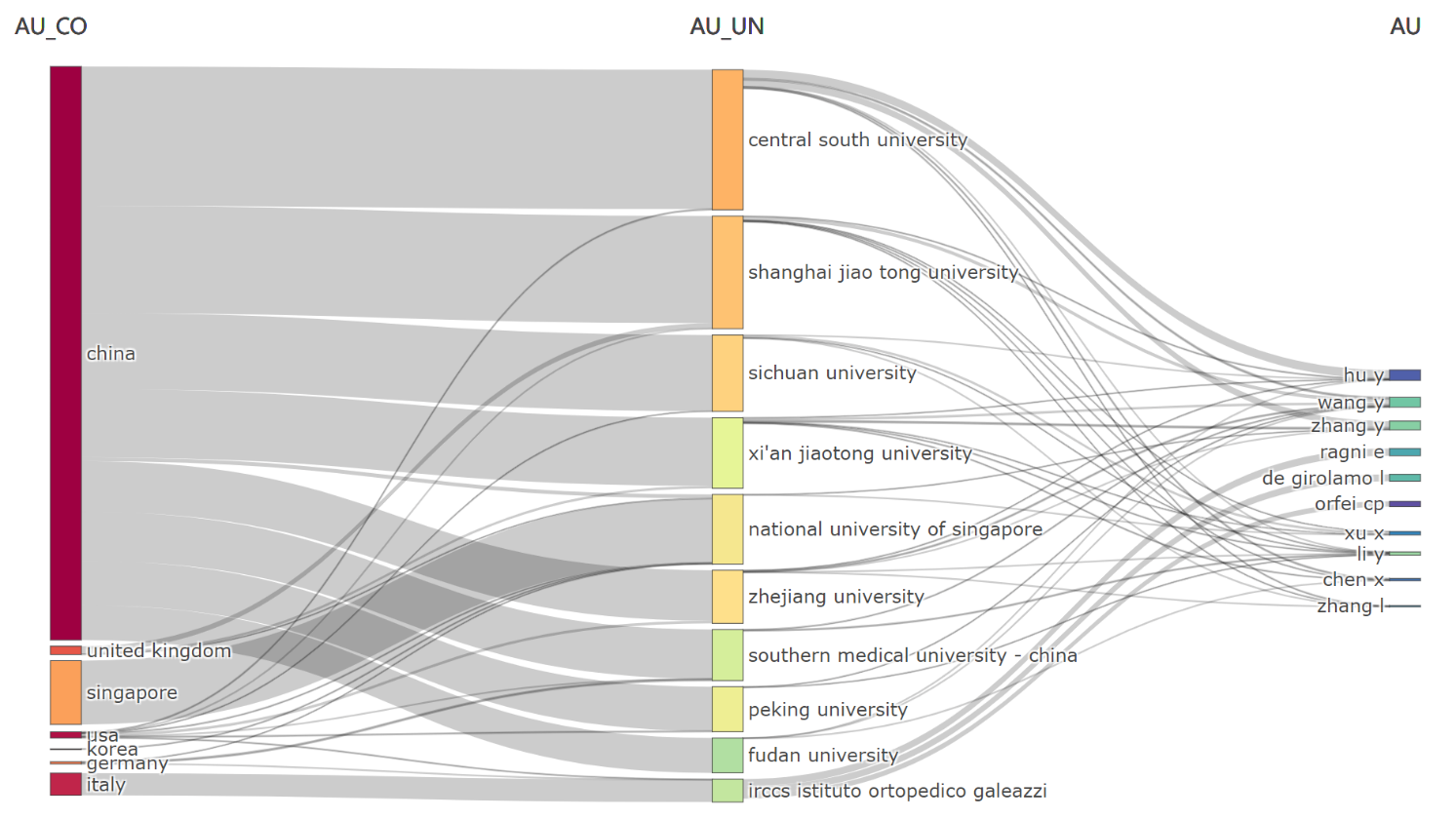


**Supplementary Figure 6.** Interconnections of the top 10 high-productivity countries/regions, institutions, and authors.
